# The Brazilian freshwater wetscape: Changes in tree community diversity and composition on climatic and geographic gradients

**DOI:** 10.1371/journal.pone.0175003

**Published:** 2017-04-10

**Authors:** Florian Wittmann, Márcia C. M. Marques, Geraldo Damasceno Júnior, Jean Carlos Budke, Maria T. F. Piedade, Astrid de Oliveira Wittmann, Juan Carlos Montero, Rafael L. de Assis, Natália Targhetta, Pia Parolin, Wolfgang J. Junk, J. Ethan Householder

**Affiliations:** 1 Department of Floodplain Ecology, Institute of Geography and Geoecology, Karlsruhe Institute for Technology, Karlsruhe, Germany; 2 MAUA Working Group, Instituto Nacional de Pesquisas da Amazônia, Manaus, Amazonas, Brazil; 3 Universidade Federal do Paraná, Curitiba, Paraná, Brazil; 4 Universidade Federal do Mato Grosso do Sul, Campo Grande, Mato Grosso do Sul, Brazil; 5 Universidade Regional Integrada do Alto Uruguai e das Missões, Erechim, Rio Grande do Sul, Brazil; 6 Universidade Federal do Amazonas, Manaus, Amazonas, Brazil; 7 Confederación de Pueblos Indígenas de Bolivia, Gobernanza de Recursos Naturales, Santa Cruz de la Sierra, Santa Cruz, Bolivia; 8 Norwegian University of Life Sciences, Ås, Akershus, Norway; 9 University of Hamburg, Biocentre Klein Flottbek, Department of Plant Diversity, Hamburg, Germany; 10 Instituto Nacional de Áreas Úmidas, Cuiabá, Mato Grosso, Brazil; 11 Botanical Research Institute of Texas, Fort Worth, Texas, United States of America; University of Oregon, UNITED STATES

## Abstract

Wetlands harbor an important compliment of regional plant diversity, but in many regions data on wetland diversity and composition is still lacking, thus hindering our understanding of the processes that control it. While patterns of broad-scale terrestrial diversity and composition typically correlate with contemporary climate it is not clear to what extent patterns in wetlands are complimentary, or conflicting. To elucidate this, we consolidate data from wetland forest inventories in Brazil and examine patterns of diversity and composition along temperature and rainfall gradients spanning five biomes. We collated 196 floristic inventories covering an area >220 ha and including >260,000 woody individuals. We detected a total of 2,453 tree species, with the Amazon alone accounting for nearly half. Compositional patterns indicated differences in freshwater wetland floras among Brazilian biomes, although biomes with drier, more seasonal climates tended to have a larger proportion of more widely distributed species. Maximal alpha diversity increased with annual temperature, rainfall, and decreasing seasonality, patterns broadly consistent with upland vegetation communities. However, alpha diversity-climate relationships were only revealed at higher diversity values associated with the uppermost quantiles, and in most sites diversity varied irrespective of climate. Likewise, mean biome-level differences in alpha-diversity were unexpectedly modest, even in comparisons of savanna-area wetlands to those of nearby forested regions. We describe attenuated wetland climate-diversity relationships as a shifting balance of local and regional effects on species recruitment. Locally, excessive waterlogging strongly filters species able to colonize from regional pools. On the other hand, increased water availability can accommodate a rich community of drought-sensitive immigrant species that are able to track buffered wetland microclimates. We argue that environmental conditions in many wetlands are not homogeneous with respect to regional climate, and that responses of wetland tree communities to future climate change may lag behind that of non-wetland, terrestrial habitat.

## Introduction

Studies in tropical biodiversity have tended to concentrate on terrestrial and marine environments, while tropical freshwater environments have been relatively overlooked [1]. Despite large knowledge gaps, tropical freshwater wetlands, including river floodplains, swamps, and gallery forests, provide critical ecosystem services and are ‘hot spots’ of biodiversity, highlighting the need for increased attention and intensified documentation of the numbers and types of organisms that inhabit them [2–3]. Furthermore, in the face of ongoing species loss due to climate change and human land- and water-use, a critical challenge is to understand the environmental and geographic patterns and causes of freshwater biodiversity [4–7].

In tropical forests, biological diversity is typically correlated with contemporary climate [8]. Among the most consistent patterns are regional correlations of rainfall or rainfall seasonality with tree diversity [9–10]. However, because the great majority of tropical tree diversity research concentrates on non-flooded, upland habitat, it remains unclear whether or not tree diversity in freshwater wetlands shows similar or contradictory patterns with climate [11]. One reason to expect differences is that wetlands temporarily store precipitation and surface runoff, and thus contrast with uplands in regards to water availability, long recognized to determine tree species distribution and, in turn, community composition and diversity [12–13]. For example, in wetlands excessive waterlogging reduces local diversity by excluding tree species intolerant to soil anoxia [14–16]. On the other hand, supplementary moisture in wetland soils can counterbalance deficits in precipitation, thus accommodating species that are otherwise sensitive to regional drought [17–19]. Wetlands are also distinguished from uplands by dynamic fluvial processes, such as river meandering. Fluvial disturbance can reduce local diversity to a handful of pioneer species [20], but can also have a positive effect by providing more opportunities for immigration, thus alleviating dispersal limitation [21–22], or by producing more environmentally variable sites for occupation, which potentially reduces competitive exclusion [23]. While many of these processes and their influence on diversity are well-understood locally, it is still unclear how they generate patterns of species diversity and distribution over large spatial scales. Understanding these large-scale patterns are, however, pertinent to broader questions of how natural communities respond to climate change, and the increasingly recognized importance of habitat heterogeneity on this response [24].

Here, we explore broad-scale patterns of tree diversity and distribution in freshwater wetlands by collating published wetland forest inventories in Brazil. We focused on Brazil for two reasons. First, the country includes 5 of the 10 largest rivers on the planet [25] and its wetlands comprise an extensive freshwater wetscape estimated to cover *c*. 20% of the national territory [26]. This territory encompasses a geographic window larger than 30 degrees latitude and longitude and spans five biomes, allowing us to examine patterns of diversity and composition along a range of climatic conditions, from savanna to rainforest and tropical to subtropical. Second, the compilation of floristic information on the wetland tree community fills a significant knowledge gap in Brazil ˗ in most Brazilian biomes, databases and species lists are well-developed for terrestrial vegetation, yet in none have wetlands been treated specifically [27–31]. More numerous than regional species lists are local accounts that compare wetland tree communities to that of nearby uplands [32–34]. However, these offer only a fragmented understanding of wetland biodiversity pattern. For example, while in the Amazon, wetland forests are mostly regarded as species-poor subsets of surrounding uplands [33,35–36], they are considered the most tree species-rich vegetation formations in drier, savanna biomes [27,37]. Such patterns call for complementary macro-scale assessments.

To further document and elucidate the processes governing tree distribution and diversity in wetland habitats and their climatic correlates across broad-spatial scales we specifically address the following questions:

1. How does the composition of wetland communities change among biomes in Brazil? Are patterns broadly similar to those of surrounding uplands?
2. How floristically diverse (gamma richness) are Brazilian wetland forests and how does this diversity compare among biomes? How does sampling effort and sample completeness compare among biomes?
3. How is regional diversity partitioned among widespread and restricted species? Are most species restricted to one biome or distributed among several, and how do biomes vary in regards to the distribution patterns of their species?
4. Is site diversity positively related to precipitation and temperature, and negatively related to seasonality and, in this sense, consistent with patterns for non-flooded, upland vegetation?

## Methods

### Biomes

**Fig 1:**
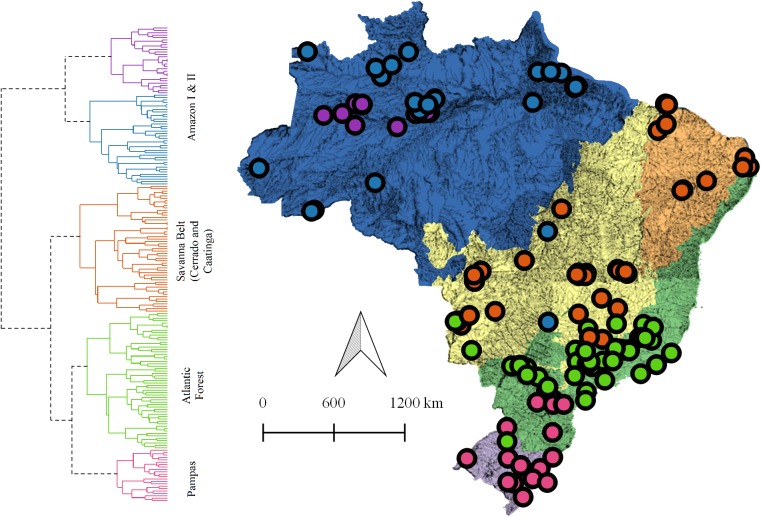
Study region, biome boundaries and site locations. Map of the five Brazilian biomes and 196 inventory sites collated in this study. Biome boundaries correspond to Veloso *et al*. [38] and are used throughout for all biome-level analyses. Point colors correspond to a hierarchal classification of sites based on a compositional dissimilarity matrix (see data analysis), and not geographic position. The Cerrado and Caatinga inventories grouped into a single cluster representing the larger Brazilian savanna belt. Mismatching colors (e.g., in Cerrado) occurs if sites do not cluster within their corresponding biome (i.e., due to differences in composition). Some sites are overlapping and not visible.

The wetland plots compiled for this study belong to all five biomes comprising the Brazilian territory: Amazon, Atlantic Forest, Cerrado, Caatinga, and Pampas (Fig 1) [38]. The Brazilian part of the Amazon covers an area of approximately 5 million km^2^. It is hot, humid and covered by evergreen tropical rainforest. Wetlands in the Amazon include large-river floodplains, riparian zones along upland streams, permanent swamps, and hydromorphic white-sand savannas [39]. Climate averages are presented in Table 1. The consecutive number of months with less than 100 mm precipitation for each site were generated with the ClimateSA v1.0 software package, available at http://tinyurl.com/ClimateSA, based on methodology described by [40]. All other climate variables were obtained from WorldClim [41].

**Table 1:** Annual climate averages for Brazilian biomes (*sensu* Veloso [38]).

**Biome**	**Consecutive months with < 100 mm precipitation**	**Mean Annual Precipitation (mm)**	**Precipitation Seasonality Index (CV)**	**Mean Annual Temperature (°C)**	**Temperature Seasonality Index (sd x 100)**
**Amazon**	2.0 (1.5)	2557 (463)	42.8 (15.1)	26.8 (0.5)	42.2 (11.4)
**Atlantic Forest**	5.11 (2.0)	1426 (208)	56.4 (22.1)	20.1 (2.1)	228.4 (35.5)
**Cerrado**	5.67 (0.8)	1337 (186)	75.7 (9.6)	23.6 (2.1)	139.1 (40.1)
**Caatinga**	8.44 (1.9)	957 (314)	92.8 (16.8)	26.1 (1.2)	105.0 (40.3)
**Pampas**	1.92 (1.5)	1440 (156)	13.6 (3.9)	18.6 (0.8)	347.0 (26.1)

Biome averages (and standard deviations) of five climate variables extracted from each site location from publicly available global climate grids. The precipitation seasonality index is calculated as the coefficient of variation (CV) of monthly precipitation values, while the temperature seasonality index is calculated as the standard deviation of mean monthly temperatures multiplied by 100.

The Atlantic Forest covers an area of approximately 1.5 million km^2^, of which approximately 12% remains under natural vegetation due to habitat destruction [42]. Climate is variable, but generally warm and wet (Table 1) [43]. Vegetation types vary, including coastal mangroves, restingas (shrub vegetation on sandy soil), evergreen tropical forests, semi-deciduous montane forests, and high altitude grasslands [44–45]. Forested freshwater wetlands include riparian forests along rivers and creeks, montane fens, bogs and swamp forests, and poorly drained depressions in coastal restingas [46].

The Cerrado covers 2 million km^2^ of which only 50% remains under natural vegetation due to modern agricultural expansion [47]. The climate is hot with a pronounced dry season (Table 1). Vegetation varies from open grasslands to closed-canopy forests. Forested wetlands include riparian forests, interfluvial depressions mostly fed by rain or groundwater (*veredas*), and hyperseasonal savannas linked to seasonal flood pulses of large rivers, such as the Pantanal and Bananal.

The semiarid Caatinga covers an area of approximately 800,000 km^2^. The biome is hot, dry and covered by seasonally dry tropical forest [48] (Table 1). Wetlands are mostly pluriannually flooded and restricted to riparian habitats or poorly drained depressions in mountain regions (*brejos de altitude*) [26].

The Pampa covers an area of approximately 180,000 km^2^ in southern, subtropical Brazil. The dominant vegetation formation is herbaceous (grass plains), while forests dominate along riparian zones, which are the most frequent freshwater wetland type.

### Data collection

We screened international and national literature on floristic inventories of Brazilian freshwater forests. Inventories were considered only if they reported species level determinations, and provided at least one quantitative measure of species abundance. We found 196 floristic inventories that fitted our inclusion criteria, totaling approximately 221 ha and 260,934 woody individuals (S1 Table). All species names were cross-checked with the TROPICOS database of the Missouri Botanical Gardens (www.tropicos.org) using the Taxonomic Name Resolution Service (http://tnrs.iplantcollaborative.org.), and synonymies, unidentified species, and species not documented in the database were excluded. Differences among biomes in species identification rates, inventory size, and inclusion criteria are summarized in Table 2.

**Table 2:** Sampling trends in Brazilian biomes (*sensu* Veloso [38]).

	Amazon	Atlantic Forest	Cerrado	Caatinga	Pampas	All Sites
**Sites (n)**	63	58	50	12	13	196
**Mean diam. cutoff ± SD (cm)**	9.07±4.56	4.70±1.32	4.62±1.52	4.3±2.21	5.36±1.54	6.10±3.51
**Area (ha)^a^**	118.69	47.2	40.65	5.94	8.9	221.37
**Individuals (n)**	83,603	91,994	58,474	8,145	18,718	260,934
**Individuals:Area (ha)^a^**	704.4	1,949	1,438.5	1,371.2	2,103.1	1,178.7
**Unident. Individuals (n)^b^**	11,639	695	1,406	157	808	14,638
**Unident. Individuals (%)^b^**	13.92	0.76	2.4	1.93	4.32	5.61
**Valid species (n)**	1,119	904	846	223	183	2,453
**Genera (n)**	385	347	352	145	120	641
**Families (n)**	80	95	91	52	48	118

^a^Some studies used abundance thresholds instead of area to reach sample size, thus area is potentially larger.

^b^Some studies indicate importance values instead of individual numbers, thus the number of unidentified individuals was estimated for these studies.

### Data analysis

#### Broad-scale composition

We examined community composition pattern across the study region using both hierarchal clustering and ordination. Both techniques require a dissimilarity matrix. Because most pair-wise dissimilarity coefficients are upward biased when sampling is partial [49], we were concerned about potential biases introduced by collating many surveys undertaken by a large number of researchers using a variety of methodologies. To account for potential differences in sampling effort we opted to use a modification of the Forbes F´ index on presence-absence data only [49]. F´ assumes that sampling is partial and is consequently a robust measure of compositional dissimilarity for incidence data obtained under a broad array of sampling conditions. Analysis of our data revealed a high correlation of F' with Simpson dissimilarity (ß_sim_, r >0.98), another popular metric for biogeographical data [50]. In using F' we guard against attributing ecological processes to explain patterns potentially driven by differences in sampling effort among surveys.

For the cluster analysis, we subjected the F´ similarity matrix to Ward’s [51] clustering algorithm and selected a number of groups which best approximated established biome boundaries [38]. For the ordination, we used both principal coordinates analysis (PCoA) and non-metric multi-dimensional scaling (NMDS) on the F´ matrix. The NMDS was optimized for two dimensions and the first dimension was rotated to the primary PCoA axis. To additionally explore compositional change with climate we mapped fitted vectors of WorldClim [41] climate variables onto both ordinations. Highly correlated (r > 0.7) WorldClim variables were removed prior to examination. All analyses were performed in R version 3.3.1 [52] using the package 'vegan' [53].

#### Regional diversity

To assess regional richness we examined species accumulation curves for each biome (*sensu* Veloso [38]). Ten random curves were computed through repeated re-sampling of sites without replacement. To account for potential systematic differences in stem density, we plotted the number of species as a function of individuals, rather than accumulated number of sites, by multiplying by the average number of stems per site in each biome [54]. For comparison, for each biome we also constructed coverage-based sampling curves from incidence data [55]. Coverage-based curves compare species richness of a set of communities by sample completeness, rather than size, where completeness is an estimate of the proportion of individuals in a community that belong to the species represented in a subsample. The R packages ‘vegan’ and ‘iNEXT’ were used for size- and coverage-based rarefactions, respectively [53, 56].

#### Local diversity

We measured alpha diversity of individual sites using Fisher’s Alpha [57]. This index is particularly suitable to our dataset because it is calculable with only two parameters, site species richness and the total number of sampled stems. While the metric is robust to differences in sample-size, we assume that species abundances conform to the lognormal distribution (i.e., few common species and many rare ones). To examine variation in local diversity and its climatic correlates we assessed change in Fisher's alpha with four relevant variables characterizing climatic conditions; these included mean annual temperature, mean annual precipitation, and measures of their seasonality [41].

Preliminary model exploration suggested that variation in Fisher’s alpha with climate is not homogeneous. Thus, model fitting was based on quantile regression to understand how the entire conditional distribution of Fisher's alpha varied with the four climate variables individually. Our use of quantile regression is consistent with the fact that numerous non-climatic hydro-edaphic factors were not available for us incorporate into models, even though they are known to strongly influence local diversity. Some of these include successional stage [22–23], habitat type [35,39], maximum flood heights and duration [58–60], and edaphic features [61–63]. The effect of these hidden ecological constraints on diversity can be examined in the rates of change of different quantiles and thus a more complete picture of the relationship between wetland diversity and environment obtained. Bootstrapping was used to estimate standard errors of coefficients for different quantiles using the R package ‘quantreg’ [64]. We additionally examined mean differences in log Fisher's alpha among biomes (*sensu* Veloso [38]) using Analysis of Variance and Tukey's Honest Significant Differences for multiple comparisons.

Worth mention are the potential sources of error in our diversity analyses that are introduced by collating work from many research teams that may use different sampling methodologies and species identification procedures. For example, the rate of unidentified individuals varies among biomes, and is highest in the Amazon region (Table 2). How this may influence alpha diversity metrics depends on a number of factors that are difficult to assess, including the local abundance of unidentified species and the accuracy of their identification. Another valid concern is geographic differences in minimum size criteria, which reflects natural physiognomic differences among forests in different biomes. For example, in the Amazon all overstory trees and the great majority of understory trees can be sampled with a 10 cm dbh (diameter at breast height) minimum size criteria, the most commonly used threshold in this region. In smaller-statured non-Amazonian forests, however, field workers often need a smaller diameter cutoff to adequately sample the understory (mostly ~5 cm).

Regional differences in methodological standards for sampling forests develop in response to natural physiognomic differences among biomes. In other words, a flexible dbh cutoff is arguably more useful if we wish to understand something about the community of mid- to overstory trees, the absolute size of which is likely to vary across such broad spatial scales. In this sense, we presume a comparison is fair. Nevertheless, we partially assessed the extent to which a strict minimum size criteria might introduce significant spatial biases in diversity analyses using an independent dataset compiled by Alwyn Gentry [65]. Gentry systematically sampled woody stems greater than 2.5cm dbh in 0.1 ha plots across a broad geographic area. Using the Gentry data we evaluated how reducing the dbh cutoff from 10 cm to 5 cm influenced Fisher’s alpha in the Amazon, the only region in our data where such large cutoffs were consistently used. Paired t-tests for 42 lowland sites (< 500 masl) indicated a moderate mean increase in Fisher’s alpha of 9.3 for smaller minimum dbh thresholds, but due to a large amount of variation this value was not statistically different from zero (t_41_ = 1.1, p = 0.26). Neither in a subset of wetland sites did we find evidence that reduced dbh cutoffs significantly alter Fisher’s alpha (t_5_ = 1.4, p = 0.22). While acknowledging potential pitfalls, we argue that the geographic trend in diameter cutoffs is not likely to have a strong statistical influence on our results and interpretations.

## Results

### Broad-scale composition

With some exceptions, the cluster analysis differentiated sites according to their corresponding biome *sensu* Veloso [38] (Fig 1). All sites located in the Amazon grouped into one of two clusters: a central Amazonian cluster (Amazon I, purple), and a second, more widespread Amazonian cluster (Amazon II, blue). Closer inspection revealed that the separation of the two Amazonian clusters is related to habitat type, with the central Amazon cluster mostly comprised of seasonal flooded, nutrient-rich white-water floodplains (várzea), and the widespread Amazon cluster mostly comprised of nutrient-poor black-water (igapó) floodplains and seasonally flooded Amazonian white-sand forests and savannas (hydromorphic campinas and campinaranas *sensu* Prance [66]). Atlantic Forest sites grouped mostly into a single cluster (green points in Fig 1), although 22% of sites were mismatched, with 10 sites clustering with the Pampas in the south (pink dots) and 3 sites clustering with the Cerrado along the complex western border with the Cerrado (orange dots). Sites located in the Pampas grouped as a single cluster, with one mismatch near the transition with the Atlantic Forest (green dot). Sites located in the Caatinga and Cerrado clustered together, reflecting the South-American belt of seasonally dry tropical forests and savannas [67–68]. Sites located in the Cerrado showed the highest rates of mismatch (25%) with groups defined by the cluster analysis. Three Cerrado sites clustered with the Amazon (blue dots) and 10 with the Atlantic Forest (green dots).

**Fig 2:**
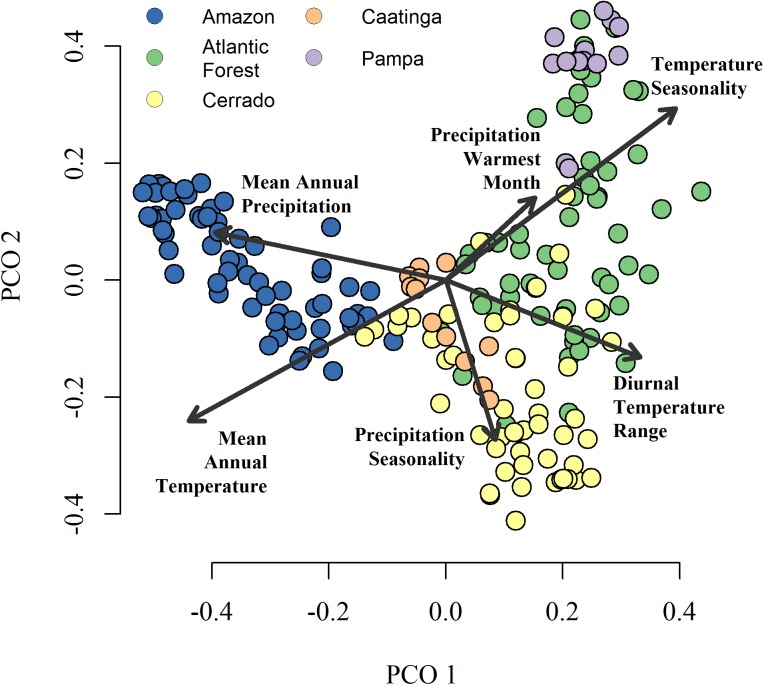
Principal coordinates ordination of vegetation assemblies in Brazilian wetlands. The principal coordinates configuration is based on a pair-wise dissimilarity matrix using Forbes F´ index. The color scheme matches biomes *sensu* Veloso [38] in Fig 1. Environmental vectors are based on WorldClim climate data [41] and show maximal correlations with the configuration. Only uncorrelated (r < 0.7), statistically significant variables are shown.

The two ordinations revealed comparable patterns of wetland composition among biomes *sensu* Veloso [38]. The first two axes of the PCoA account for 13.3 and 7.5% of compositional variation (Fig 2). The first axis contrasts Amazonian sites from all others, and is associated with variation in annual temperature and precipitation. The secondary axis contrasts sites among non-Amazonian biomes and is associated with variation in temperature and precipitation seasonality. The NMDS (stress = 0.17) shows a similar configuration to the PCoA, although Caatinga sites have a more outlying distribution in accordance with their distinct vegetation communities (S1 Fig). In both ordinations Amazonian wetlands are associated with higher annual temperature and precipitation, Cerrado and Caatinga wetlands with higher precipitation seasonality, and Atlantic Forest and Pampas with high temperature seasonality.

### Regional diversity

**Fig 3:**
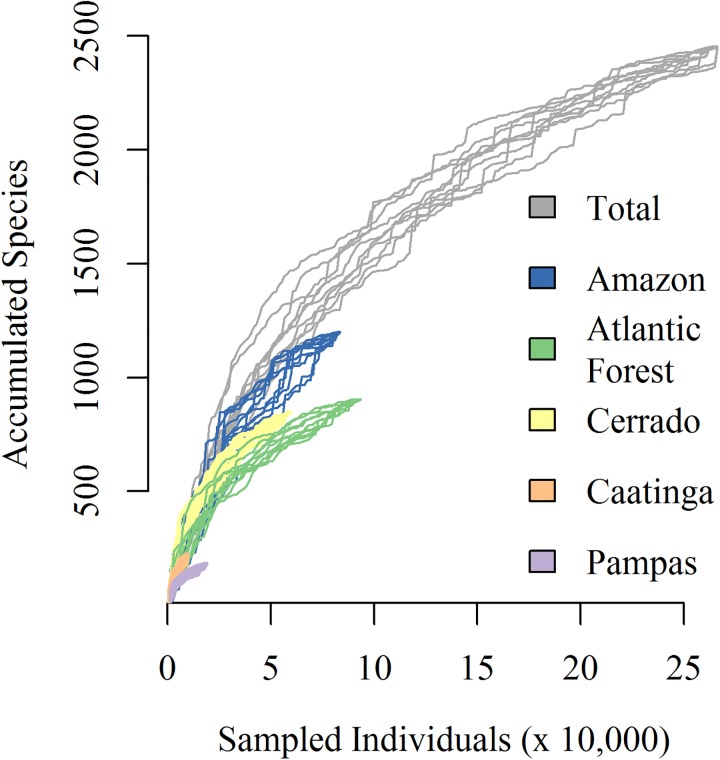
Regional wetland diversity in Brazilian biomes (*sensu* Veloso [38]). Random curves were generated by repeated re-sampling of pooled sites within biomes (colored curves) or all sites combined (grey curve). The x-axis is rescaled to the number of individuals, based on the average number of stems per site of each biome.

In total, the 196 wetland inventories revealed 2,453 woody species with valid names. These were distributed among 641 genera and 118 families. The Amazon had the greatest number of documented species occurring in wetlands (1,199) followed by the Atlantic Forest (904), Cerrado (846), Caatinga (223) and Pampas (183). Species accumulation curves showed that sampling effort differs markedly among biomes, especially in the Caatinga and Pampas where fewer wetland inventories were available (Fig 3). Coverage-based sampling curves ranked biomes similarly in regards to richness, with the Amazon ~1.5 times more rich in species than either the Cerrado or Atlantic Forest for a given coverage reference level (S2 Fig). Estimates of sampling completeness among biomes revealed the largest coverage deficit (given as 1- estimated biome coverage) for Caatinga (0.25), indicating a one in four chance that a new individual sampled will be a previously unsampled species. These chances decrease to less than one in ten for the Atlantic Forest, the biome with the smallest coverage deficit.

Of the 2,453 tree species, only four (0.16%) occur in all biomes [*Casearia sylvestris* (Salicaceae), *Sapium glandulosum* (Euphorbiaceae), *Cedrela fissilis* (Meliaceae) and *Handroanthus heptaphyllus* (Bignoniaceae)], whereas 1,786 tree species (72.8%) have exclusive occurrence in one of the five biomes. The Amazon showed the highest proportion of exclusive species, followed distantly by the Atlantic Forest, Caatinga, Cerrado, and Pampas (Table 3). Similarly, the Amazon wetlands have the highest number of exclusive genera (40.5%), followed distantly by the Caatinga (15.2%), Atlantic Forest (15%), Cerrado (11.1%) and Pampas (6.7%). A complete species list including overall biome and country-wide species frequencies is presented in S2 Table. The site x species data in three-column format is made available in S1 Dataset.

**Table 3:** For each biome, the number (and proportion) of tree species occurring in one, two, or more biomes (*sensu* Veloso [38]).

**Number of Biomes**	**Amazon**	**Atlantic Forest**	**Cerrado**	**Caatinga**	**Pampas**
**Exclusive species**	987 (0.82)	382 (0.42)	292 (0.35)	91 (0.41)	34 (0.19)
**in 2 biomes**	116 (0.10)	341 (0.38)	370 (0.44)	58 (0.26)	71 (0.39)
**in 3 biomes**	64 (0.05)	139 (0.15)	144 (0.17)	38 (0.17)	56 (0.31)
**in 4 biomes**	28 (0.02)	38 (0.4)	36 (0.04)	32 (0.14)	18 (0.10)
**in 5 biomes**	4 (0.003)	4 (0.004)	4 (0.005)	4 (0.02)	4 (0.02)
**Total species**	**1199**	**904**	**846**	**223**	**183**

### Local diversity

**Fig 4:**
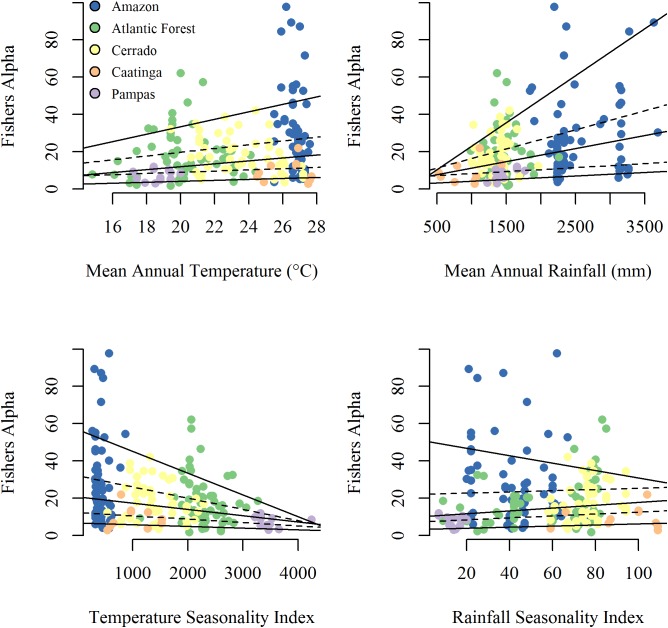
Variation in Fisher’s alpha along four climate gradients. Quantile regression fits are indicated with solid (tau = 0.1, 0.5, and 0.9) and dashed lines (tau = 0.3, 0.7). The color scheme matches biome colors in Fig 1.

Quantile regression plots showed that the dispersion of alpha diversity increases in warmer, wetter and less seasonal climates (Fig 4). Thus, both the highest alpha diversities, as well as amongst the lowest, are observed in wetlands of wet and warm climates. Positive diversity relationships with mean annual rainfall and temperature, and negative relationships with rainfall and temperature seasonality were only revealed at the highest diversity values associated with the uppermost quantiles. For example, the rate of change in diversity at the 90^th^ quantiles are 7- to 14-fold greater than those of the 10^th^. Shallow slopes and narrow spacing of the lower quantile regression lines indicate that the conditional distribution of diversity is highly right-skewed and, for the majority of wetland sites, exhibits weak association to the studied climate variables. Indeed, for all investigated climate variables the coefficient estimates for the first third to four fifths of quantiles were not statistically different from zero (S3 Fig). Finally, estimated empirical quantile functions of diversity for wetlands at the 10^th^ and 90^th^ percentile of the sampled climate distributions revealed that expected modal values of Fisher’s alpha are quite similar, regardless of large climate differences (S4 Fig).

**Fig 5:**
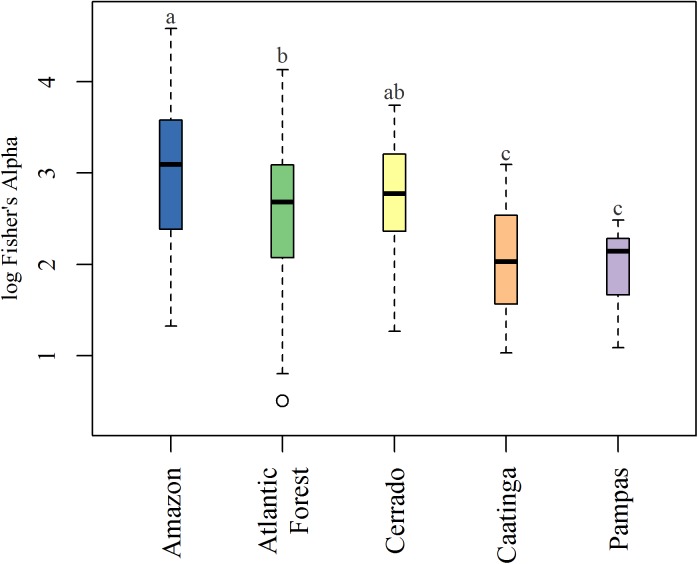
Comparison of log Fisher's alpha among biomes. Significant differences were assessed using Tukey's Honest Significant Difference, with letters indicating group differences.

When assessed among biomes, Amazon wetland plots showed the highest mean tree alpha diversity (28.51±22.87), followed by wetlands of the Cerrado (17.97±9.97), the Atlantic Forest (17.63±13.1), Caatinga (9.38±5.85) and Pampas (7.5±2.99). However, mean values in the large, forested biomes, Amazon and Atlantic Forest, are markedly influenced by few plots with high diversity values (S5 Fig). Using the log of Fisher's alpha to compare mean diversity among biomes there is strong evidence for differences (F_4,188_ = 8.8, p < 0.001) (Fig 5). A Tukey multiple-comparison test indicated these differences were largely between the small (Caatinga and Pampas) and larger biomes (Amazon, Atlantic Forest and Cerrado). Among the three larger biomes, mean differences in Fisher's alpha were *not* detected between Cerrado and Amazon wetlands, nor between Cerrado and Atlantic Forest wetlands.

## Discussion

The data indicate that wetland forest communities do show parallels with upland, non-flooded vegetation. First, composition of wetland plant assemblages differs from one biome to another and along climatic gradients. Second, more humid and larger biomes nearer the equator had more tree species, consistent with well-known richness relationships with area, latitude, temperature and precipitation [10,69–71]. Lastly, maximal alpha diversity was strongly associated with climate–forested wetland sites in warm and humid biomes (i.e., the Amazon and Atlantic Forest) had the highest maximal Fisher's diversities. These three general patterns are interrelated, and taken together suggest that in similar fashion to upland forests, 1) wetland forest trees are recruited from regionally distinct species pools, 2) the composition and richness of these pools are the result of historical processes that operate at large spatial scales (e.g., speciation, extinction, and biogeographic dispersal), and 3) these processes can trickle down to affect local tree diversity in wetlands [72].

While on the surface wetlands do conform to upland vegetation pattern, the magnitude of change along diversity gradients was much less than we expected. For example, the Brazilian savanna belt (i.e., Caatinga and Cerrado with 959 species) has 18 tree species more in wetlands than southern tropical-subtropical forested biomes combined (i.e., Atlantic Forest and Pampas with 941 species), despite ~44,000 fewer individuals sampled. Moreover, species accumulation and coverage-based sampling curves predict higher rates of previously unsampled species for additional sites in wetlands of dry regions, and especially in Caatinga. In another example, strong associations between maximal alpha diversity and climate were driven by only a small proportion of high-diversity plots in species-rich regions. In regions with optimal climates for tree growth (i.e., Amazonia and Atlantic Forest) the diversity index for the majority of plots was *lower* than high-diversity plots in semi-arid savanna biomes, Caatinga and Cerrado. In a related result, quantile regression models showed only slight variation in estimated modal alpha diversity despite substantial climate differences (S4 Fig). Finally, when mean site diversity was compared among biomes, Cerrado wetlands were as diverse as those of the Amazon and Atlantic Forest, demonstrating that wetlands of savanna biomes are not necessarily less diverse, on average, than those of tree species rich, forested biomes (Fig 5).

### Insights into wetland community ecology

The great majority of wetland diversity research has focused on the local causes of diversity (e.g., flooding duration, height, water chemistry, and disturbance). Based on this line of inquiry, it is clear that multiple abiotic stressors in wetlands affect establishment, growth and survival of the great majority of trees [16,35,73]. The magnitude of the effect on diversity that these small-scale processes have is large [14], and may partially explain why we observed, for most sites, poor association of wetland tree diversity with climate variables.

However, a more complete understanding of the causes of wetland alpha diversity might be gained by considering not only the small-scale processes, which generally limit the number of species able to colonize and survive in wetlands, but also the large-scale processes, which generally determine the size of the species pool available for colonization [72]. Such regional species pools are in part governed by factors that drive broad-scale distributions of species, of which climate is typically a good predictor [74]. However, one fundamental distinction between uplands and wetlands is water, which can both attenuate temperature fluctuations due to a high latent heat capacity [17,75] and ameliorate regional drought due to high soil moisture availability [18,76]. Cooler and moister wetland microclimates can potentially compensate otherwise unsuitable regional conditions, with potentially large effects on species broader distributions and, in turn, the size of the regional species pool available for colonization. For example, to the extent that drought-sensitive species are capable of tracking buffered wetland conditions, small differences in wetland soil moisture availability may extend species ranges into regions with higher evapotranspiration demands. In this way, wetland diversity in relatively species-poor dry-seasonal climates (e.g., Cerrado) may be propped up by biogeographic immigration.

One pattern in our data that is consistent with these predictions is the higher rate of cluster-biome mismatch in the arid Cerrado with more humid Amazonian or Atlantic Forest clusters (Fig 1). This pattern might be explained by encroaching arid-edge populations of species from the more humid biomes into small areas of Cerrado wetland habitat, where they are able to meet moisture requirements outside their core geographic ranges. If interpretable as such, these drought-sensitive immigrants may bolster high wetland diversity in the Cerrado. According to our data, 65% of Cerrado wetland species also occurred in at least one other biome, the highest rate among the three large biomes. Even this figure, however, may be an underestimate if most species are not restricted to wetland habitat, a probable scenario (Table 3). For example, many species documented in Cerrado wetlands likely occur in non-wetland habitats of neighboring humid biomes, but are erroneously counted as exclusives in our wetlands dataset. Thus, our estimate of the contribution of biogeographic immigrants from more humid biomes to Cerrado wetland diversity is still likely conservative, but largely consistent with previous studies [77].

The Caatinga, another semi-arid savanna biome, contrasts with the Cerrado in its consistently low alpha diversity and a relatively unique flora—41% of Caatinga species are exclusive to this biome. The drier climate of Caatinga may exceed the buffering capacity required for many drought-sensitive immigrants. Alternatively, factors that hinder successful immigration may reduce local diversity. For example, lower precipitation and runoff in Caatinga results in smaller, more ephemeral wetlands that support smaller drought-intolerant tree populations more susceptible to local extinction. Also, Caatinga lacks direct fluvial corridors to forested biomes that potentially enhance migration of drought-sensitive species. Such corridors are absent in the Caatinga, but prevalent in the Cerrado where large Amazonian rivers such as the Araguaia, Tocantins, and Xingu provide direct fluvial connection to moister, more species-rich regions.

At this point we can only offer hypotheses to frame future investigation. Based on our findings, we predict that (i) for many tree species, the strength of wetland habitat association is linked to climate, and may be greater towards the limits of species’ climatic ranges where a wetland buffering effect is more important for local persistence; (ii) for communities, the relative strengths of local and regional causes of wetland diversity vary with climate, and that in more seasonal climates, wetland tree diversity is propped up by a larger regional effect (i.e., biogeographic immigration).

### Implications: Wetlands and climate change

Wetlands are thought to have played major roles as climate refugia for tree species during past climate fluctuations throughout much of the evolutionary history of Neotropical biomes, particularly in drought-prone regions [18,76]. While ongoing climate change is predicted to increase annual precipitation for some of our study region, including parts of Pampas and Atlantic forest, climate change predictions throughout northern Brazil, and especially in the Amazon, include higher temperatures, more severe dry seasons, and possibly reduced annual rainfall [78]. The combined effects are expected to greatly reduce available moisture for plant growth in one of the most tree species-rich regions on the planet; some even warn of widespread regional die-back and biotic attrition [79,80].

A promising way of predicting how drought-sensitive tree communities might respond to future, drier conditions is to examine how they react where current climate is already drier. Our analyses suggest that wetland habitats can sustain local and even regional diversity across a broad range of climates. Based on this, we have argued that wetland environments are not homogeneous with respect to regional climate and, because of this, the response of wetland tree diversity to climate change may lag behind non-flooded terrestrial habitats. In this sense, colonization of wetland habitat may be an important response of trees in the face of future climate adversity in the Amazon region, playing a role in both accommodating populations of drought-sensitive species *in situ* as well as facilitating species range adjustments [81]. Under such circumstances, we would expect wetlands to increasingly sustain a growing compliment of regional diversity. Their capacity to support this diversity under continued wetland degradation through pollution, unsustainable resource use, deforestation and river damming for hydropower is, however, not clear [3,82].

## Supporting information

S1 Table List of 196 freshwater wetland tree inventories collated for this study. Biome *sensu* Veloso [38] is indicated by code (AM = Amazon, AF = Atlantic Forest, CERR = Cerrado, CAAT = Caatinga, PM = Pampas). The number of individuals and species were taken directly from each publication and used to calculate Fisher’s Alpha.
(DOCX)

S2 Table Species checklist for wetland trees in Brazil. Author names and synonymies were checked using the taxonomic name resolution service. Species frequencies are given for each biome.
(XLSX)

S1 Dataset Species x site data in three-column format. (XLSX)

S1 Fig Non-metric multidimensional scaling of wetland vegetation communities. The solution was optimized for two dimensions and rotated to principal components (see Fig 2). The main distinction with the principal components configuration is the relative position of Caatinga sites, which are more outlying in the NMDS. Fitted vectors are of WorldClim climate data [41].
(TIFF)

S2 Fig Coverage-based species accumulation curves for each biome seperately and the combined dataset. We used the function iNEXT from the ‘iNEXT’ package [56] with the settings iNEXT(x, q = 0, datatype = “incidence_freq”, conf = 0.95).
(TIFF)

S3 Fig Quantile regression coefficients, intercepts, and 95% confidence intervals for all quantiles. Generally, coefficients are nearly zero for the lower to mid quantiles of the distribution, but rapidly increase at the highest quantiles. The pattern indicates strong climate association only at maximal wetland tree diversity, driven by relatively few sites.
(TIF)

S4 Fig Density functions of diversity at opposite climate extremes. Estimated density functions are based on the corresponding quantile regression solutions (see Fig 4) for all values of tau in 0 to 1. Estimates are presented for the 10th and 90th quantiles of each climate variable, as specified in the legend insets. Mean annual temperature is multiplied by 10, as in the original WorldClim database [41].
(TIFF)

S5 Fig Distribution of Fisher’s alpha values in the investigated biomes, *sensu* Veloso [38]. Median values are indicated with a thick vertical line. Note that median values are relatively similar for the three best-sampled biomes (Amazon, Atlantic Forest, and Cerrado).
(TIFF)
