# Supplementary material for: The Brazilian freshwater wetscape: Changes in tree community diversity and composition on climatic and geographic gradients
Source: PLoS One. 2017 Apr 10;12(4):e0175003. doi: 10.1371/journal.pone.0175003 (PMC5386251; doi:10.1371/journal.pone.0175003)
Supplement: S1 Table — Biome sensu Veloso [38] is indicated by code (AM = Amazon, AF = Atlantic Forest, CERR = Cerrado, CAAT = Caatinga, PM = Pampas). The number of individuals and species were taken directly from each publication and used to calculate Fisher’s Alpha. (DOCX) [file pone.0175003.s001.docx]

**S1 Table. List of 196 freshwater wetland tree inventories collated for this study.** Biome is indicated by code (AM = Amazon, AF = Atlantic Forest, CERR = Cerrado, CAAT = Caatinga, PM = Pampas). The number of individuals and species were taken directly from each publication and used to calculate Fisher’s Alpha.

| Publication | Biome | State | Lat./Long. | River - basin | Ecosystem or habitat type | Minimum dbh (cm) threshold | Area (ha) | No. of Individuals | No. of Species |
| --- | --- | --- | --- | --- | --- | --- | --- | --- | --- |
| Santos & Jardim (2006) *Acta Amazônica* 36: 437-446. | AM | Pará | 01°13’S/48°17’W | Aracy - Amazon | Várzea | 10 | 4.0 | 3286 | 70 |
| Gama *et al.* (2002) *R Árvore* 26: 559-566. | AM | Pará | 00°09’S/50°23’W | Amazon | Várzea | 0-15 | 0.25 | 3345 | 63 |
| Gama *et al*. (2003) *Ciência Florest* 13: 71-82. | AM | Pará | 00°09’S/50°23’W | Amazon | Várzea | 0-15 | 0.29 | 8981 | 70 |
| Carim *et al*. (2008) *Sci Fo. Piracicaba* 36: 191-201. | AM | Amapá | 00°06’S/51°17’W | Mazagão - Amazon | Várzea | 10 | 5.0 | 2068 | 82 |
| Almeida *et al*. (2004) *Acta Amazônica* 34: 513-524. | AM | Pará | 02°38’S/51°56’W | Xingu - Amazon | Várzea | 10 | 1.0 | 676 | 42 |
| Almeida *et al*. (2004) *Acta Amazônica* 34: 513-524. | AM | Pará | 01°26’S/48°39’W | Pará - Amazon | Várzea | 10 | 1.0 | 735 | 78 |
| Almeida *et al.* (2004) *Acta Amazônica* 34: 513-524. | AM | Pará | 00°15’S/49°39’W | Amazon | Várzea | 10 | 1.0 | 809 | 36 |
| Almeida *et al*. (2004) *Acta Amazônica* 34: 513-524. | AM | Pará | 00°05’S/50°20’W | Amazon | Várzea | 10 | 1.0 | 691 | 60 |
| Cattanio *et al.* (2002) *Rev Bras Bot*  25: 419-430. | AM | Pará | 01°20’S/48°30’W | Guamá - Amazon | Várzea | 5 | 0.2 | 504 | 45 |
| Cattanio *et al*. (2002) *Rev Bras Bot* 25: 419-430. | AM | Pará | 01°20’S/48°30’W | Guamá - Amazon | Várzea | 5 | 0.2 | 238 | 67 |
| Cattanio *et al.* (2002) *Rev Bras Bot* 25: 419-430. | AM | Pará | 01°20’S/48°30’W | Guamá - Amazon | Várzea | 5 | 1.0 | 588 | 84 |
| Pires & Koury (1959) *Bol Técnico IAN* 36: 3-44. | AM | Pará | 01°20’S/48°30’W | Guamá - Amazon | Várzea | 5-8 | 1.0 | 539 | 53 |
| Pires & Koury (1959) *Bol Técnico IAN* 36: 3-44. | AM | Pará | 01°20’S/48°30’W | Guamá - Amazon | Várzea | 10 | 3.8 | 1837 | 107 |
| Black *et al*. (1950) *Bot Gazette* 111: 413-425. | AM | Pará | 01°20’S/48°30’W | Guamá - Amazon | Várzea | 10 | 3.8 | 564 | 62 |
| Klinge *et al*. (1996) *Acta Amazônica* 25: 201-220. | AM | Amazonas | 03°15’S/59°58’W | Solimôes - Amazon | Várzea | 5 | 0.9 | 1086 | 43 |
| Wittmann *et al*. (2002) *J Trop Ecol* 18: 805-820. | AM | Amazonas | 03°18’S/60°00’W | Solimôes - Amazon | Várzea | 10 | 0.88 | 380 | 68 |
| Wittmann *et al*. (2002) *J Trop Ecol* 18: 805-820. | AM | Amazonas | 03°15’S/59°58’W | Solimôes - Amazon | Várzea | 10 | 1.0 | 466 | 35 |
| Worbes *et al.* (1992) *J Veg Sci* 3: 553-564. | AM | Amazonas | 03°15’S/59°58’W | Solimôes - Amazon | Várzea | 5 | 0.76 | 1217 | 35 |
| Worbes *et al.* (1992) *J Veg Sci* 3: 553-564. | AM | Amazonas | 03°08’S/59°50’W | Solimôes - Amazon | Várzea | 10 | 0.4 | 745 | 27 |
| Worbes *et al.* (1992) *J Veg Sci* 3: 553-564. | AM | Amazonas | 03°15’S/59°58’W | Solimôes - Amazon | Várzea | 5 | 0.21 | 800 | 30 |
| Worbes *et al.* (1992) *J Veg Sci* 3: 553-564. | AM | Amazonas | 03°25’S/60°46’W | Solimôes - Amazon | Várzea | 5 | 1.0 | 627 | 89 |
| Worbes *et al.* (1992) *J Veg Sci* 3: 553-564. | AM | Amazonas | 03°15’S/59°40’W | Solimôes - Amazon | Várzea | 10 | 0.4 | 560 | 41 |
| Revilla (1991) PhD-Thesis INPA, Manaus – Brazil. | AM | Amazonas | 03°15’S/59°58’W | Solimôes - Amazon | Várzea | 5 | 1.0 | 947 | 48 |
| Revilla (1991) PhD-Thesis INPA, Manaus – Brazil. | AM | Amazonas | 03°23’S/60°40’W | Solimôes - Amazon | Várzea | 5 | 5.0 | 730 | 129 |
| Revilla (1991) PhD-Thesis INPA, Manaus – Brazil. | AM | Amazonas | 03°25’S/60°46’W | Solimôes - Amazon | Várzea | 5 | 10.0 | 901 | 187 |
| Assis *et al.* (2015) *J Veg Sci* 26: 312-322. | AM | Amazonas | 04°24’S/61°88’W | Purus - Amazon | Várzea | 10 | 3.0 | 1951 | 135 |
| Assis *et al.* (2015) *J Veg Sci* 26: 312-322. | AM | Amazonas | 03°23’S/66°05’W | Juruá - Amazon | Várzea | 10 | 3.0 | 1844 | 147 |
| Assis *et al.* (2015) *J Veg Sci* 26: 312-322. | AM | Amazonas | 03°37’S/67°48’W | Jutai - Amazon | Várzea | 10 | 3.0 | 2261 | 146 |
| Ayres (1993) Soc. Civil Mamirauá, Brasilia. | AM | Amazonas | 02°58’S/64°55’W | Japurá - Amazon | Várzea | 10 | 1.0 | 580 | 135 |
| Ayres (1993) Soc. Civil Mamirauá, Brasília. | AM | Amazonas | 02°58’S/64°55’W | Japurá - Amazon | Várzea | 10 | 1.0 | 460 | 109 |
| Queiroz (1995) Soc. Civil Mamirauá, Brasília. | AM | Amazonas | 02°58’S/64°55’W | Japurá - Amazon | Várzea | 10 | 1.0 | 430 | 68 |
| Queiroz (1995) Soc. Civil Mamirauá, Brasília. | AM | Amazonas | 02°58’S/64°55’W | Japurá - Amazon | Várzea | 10 | 1.0 | 443 | 37 |
| Wittmann *et al*. (2002) *J Trop Ecol* 18: 805-820. | AM | Amazonas | 02°51’S/64°55’W | Japurá - Amazon | Várzea | 10 | 1.0 | 641 | 45 |
| Wittmann *et al*. (2002) *J Trop Ecol* 18: 805-820. | AM | Amazonas | 02°51’S/64°55’W | Japurá - Amazon | Várzea | 10 | 1.0 | 434 | 91 |
| Wittmann & Junk (2003) *J Biogeography* 30: 1533-1544. | AM | Amazonas | 02°51’S/64°55’W | Japurá - Amazon | Várzea | 5 | 0.18 | 611 | 134 |
| Assis & Wittmann (2011) *Flora* 206: 251-260. | AM | Amazonas | 02°51’S/64°55’W | Japurá - Amazon | Várzea | 5 | 0.31 | 1486 | 116 |
| Wittmann *et al*. (2002) *J Trop Ecol* 18: 805-820. | AM | Amazonas | 02°51’S/65°02’W | Japurá - Amazon | Várzea | 10 | 1.0 | 434 | 158 |
| Schöngart (2003) PhD-Thesis Univ. of Göttingen – Germany. | AM | Amazonas | 02°53’S/64°53’W | Japurá - Amazon | Várzea | 10 | 1.0 | 838 | 30 |
| Schöngart (2003) PhD-Thesis Univ. of Göttingen – Germany. | AM | Amazonas | 02°54’S/64°53’W | Japurá - Amazon | Várzea | 10 | 1.0 | 487 | 45 |
| Schöngart (2003) PhD-Thesis Univ. of Göttingen – Germany. | AM | Amazonas | 02°47’S/65°04’W | Japurá - Amazon | Várzea | 10 | 1.0 | 504 | 87 |
| Schöngart (2003) PhD-Thesis Univ. of Göttingen – Germany. | AM | Amazonas | 02°51’S/64°55’W | Japurá - Amazon | Várzea | 10 | 1.0 | 462 | 111 |
| Morais (2010) Monografia Univ. Federal Rural do Rio de Janeiro – Brazil. | AM | Acre | 10°57’S/68°19’W | Acre - Amazon | Várzea | 20 | 3.0 | 371 | 88 |
| Morais (2010) Monografia Univ. Federal Rural do Rio de Janeiro – Brazil. | AM | Acre | 10°66’S/68°40’W | Acre - Amazon | Várzea | 20 | 3.0 | 656 | 140 |
| Campbell *et al*. (1992) *Biol J Linn Soc* 108: 213-237. | AM | Acre | 07°38’S/72°40’W | Juruá - Amazon | Várzea | 10 | 1.0 | 523 | 106 |
| Campbell *et al*. (1992) *Biol J Linn Soc* 108: 213-237. | AM | Acre | 07°38’S/72°40’W | Juruá - Amazon | Várzea | 10 | 1.0 | 420 | 73 |
| Campbell *et al*. (1992) *Biol J Linn Soc* 108: 213-237. | AM | Acre | 07°38’S/72°40’W | Juruá - Amazon | Várzea | 10 | 1.0 | 777 | 20 |
| Targhetta *et al*. (2015) *Folia Geobot* 50: 185-205. | AM | Amazonas | 02°11’S/59°01’W | Uatumã - Amazon | Campina | 10 | 1.0 | 560 | 84 |
| Targhetta *et al*. (2015) *Folia Geobot* 50: 185-205. | AM | Amazonas | 02°11’S/59°01’W | Uatumã - Amazon | Campina | 10 | 1.0 | 503 | 46 |
| Targhetta *et al*. (2015) *Folia Geobot* 50: 185-205. | AM | Amazonas | 02°10’S/59°01’W | Uatumã - Amazon | Campina | 10 | 1.0 | 786 | 67 |
| Targhetta *et al*. (2015) *Folia Geobot* 50: 185-205. | AM | Amazonas | 02°15’S/59°02’W | Uatumã - Amazon | Igapó | 10 | 1.0 | 540 | 50 |
| Targhetta *et al*. (2015) *Folia Geobot* 50: 185-205. | AM | Amazonas | 02°14’S/59°00’W | Uatumã - Amazon | Igapó | 10 | 1.0 | 927 | 33 |
| Targhetta *et al*. (2015) *Folia Geobot* 50: 185-205. | AM | Amazonas | 02°15’S/59°00’W | Uatumã - Amazon | Igapó | 10 | 1.0 | 695 | 26 |
| Assis *et al.* (2015) *J Veg Sci* 26: 312-322. | AM | Amazonas | 04°12’S/65°08’W | Tefé - Amazon | Igapó | 10 | 3.0 | 1750 | 195 |
| Parolin *et al*. (2004) *Amazoniana* 18: 29-47. | AM | Amazonas | 03°02’S/60°17’W | Negro | Igapó | 3.2 | 0.1 | 162 | 44 |
| Parolin *et al.* (2004) *Amazoniana* 18: 29-47. | AM | Amazonas | 03°02’S/60°17’W | Negro | Igapó | 32 | 1.5 | 43 | 10 |
| Stropp *et al.* (2011) *Acta Amazônica* 41: 521-544. | AM | Amazonas | 01°48’N/68°71’W | Içana, Negro | Campina | 10 | 4.0 | 2524 | 290 |
| Montero *et al.* (2014) *Hydrobiologia* 729: 229-246. | AM | Amazonas | 02°37’S/60°53’W | Negro | Igapó | 10 | 3.0 | 1371 | 102 |
| Damasco *et al*. (2013) *J. Veg. Sci.* 24: 384-394. | AM | Roraima | 01°46’N/61°02’W | Viruá – Negro | Campina | 1 | 17.0 | 16599 | 303 |
| Montero *et al.* (2014) *Hydrobiologia* 729: 229-246. | AM | Amazonas | 00°41’S/63°09’W | Negro | Igapó | 10 | 2.25 | 1734 | 88 |
| Montero *et al.* (2014) *Hydrobiologia* 729: 229-246. | AM | Amazonas | 00°23’S/63°50’W | Negro | Igapó | 10 | 3.0 | 1577 | 108 |
| Montero *et al.* (2014) *Hydrobiologia* 729: 229-246. | AM | Amazonas | 00°46’S/62°29’W | Juvaris, Negro | Igapó | 10 | 1.75 | 1444 | 83 |
| Brito (2010) Master-Diss. Instituto Nacional de Pesquisas da Amazônia, Manaus-Brazil. | AM | Amazonas | 02°55’S/59°53’W | Igarapé Barro Branco - Amazon | Riparian | 10 | 2.00 | 1378 | 246 |
| Santos (2007) Monografia Fundação Univ. Federal de Rondônia, Porto Velho-Brazil. | AM | Rondônia | 08°49’S/63°56’W | Igarapé Mato Grosso – Madeira | Riparian | 3.2 | 0.5 | 1077 | 243 |
| Arieira & Cunha (2006) *Acta Bot Bras* 20: 569-580. | CERR | Mato Grosso | 16°00’S/56°00’W | Cuiabá - Paraguai | Pantanal | 5 | 1.0 | 1050 | 58 |
| Arieira & Cunha (2006) *Acta Bot Bras* 20: 569-580. | CERR | Mato Grosso | 16°00’S/56°00’W | Cuiabá - Paraguai | Pantanal | 5 | 1.0 | 742 | 19 |
| Arieira & Cunha (2006) *Acta Bot Bras* 20: 569-580. | CERR | Mato Grosso | 16°00’S/56°00’W | Cuiabá - Paraguai | Pantanal | 5 | 1.0 | 441 | 29 |
| Arieira & Cunha (2006) *Acta Bot Bras* 20: 569-580. | CERR | Mato Grosso | 16°00’S/56°00’W | Cuiabá - Paraguai | Pantanal | 5 | 1.0 | 916 | 26 |
| Wittmann *et al.* (2008) *Folia Geobot* 43: 397-411. | CERR | Mato Grosso do Sul | 19°34’S/57°01’W | Miranda - Paraguai | Pantanal | 10 | 1.0 | 443 | 46 |
| Corsini & Guarim-Neto (2000) III Simpósio Recursos Naturais e sócio-econômicos do Pantanal. Univ. Federal de Mato Grosso, Cuiabá – Brazil. | CERR | Mato Grosso | 15°51’S/56°04’W | Cuiabá - Paraguai | Pantanal | 3.2 | - | 200 | 19 |
| Damasceno-Junior *et al*. (2005) *Flora* 200: 119-135. | CERR | Mato Grosso do Sul | 19°01’S/57°27’W | Paraguai | Pantanal | 4.8 | 1.06 | 695 | 37 |
| Fonseca *et al*. (2004) IV Simpósio Recursos Naturais e sócio-econômicos do Pantanal. Univ. Federal de Mato Grosso, Cuiabá – Brazil. | CERR | Mato Grosso | 15°51’S/56°04’W | Cuiabá-Paraguai | Pantanal | 3.2 | 0.4 | 142 | 18 |
| Salis *et al*. (2004) *Rev Bras Bot* 27: 671-684. | CERR | Mato Grosso do Sul | 19°05’S/57°47’W | Paraguai | Pantanal | 2.9 | - | 80 | 32 |
| Silva et al. (2000) III Simpósio Recursos Naturais e sócio-econômicos do Pantanal. Univ. Federal de Mato Grosso, Cuiabá – Brazil. | CERR | Mato Grosso do Sul | 18°59’S/56°39’W | Paraguai | Pantanal | 3 | 0.05 | 200 | 41 |
| Silva et al. (2000) III Simpósio Recursos Naturais e sócio-econômicos do Pantanal. Univ. Federal de Mato Grosso, Cuiabá – Brazil. | CERR | Mato Grosso do Sul | 18°59’S/56°39’W | Paraguai | Pantanal | 3 | 0.05 | 200 | 37 |
| Ferreira (2010) Master-Diss. Univ. Federal de Brasília – Brazil. | CERR | Minas Gerais | 17°28’S/46°33’W | Paracatu – São Francisco | Riparian | 5 | 1.0 | 697 | 70 |
| Felfili (1995) *J Trop Ecol* 11: 67-83. | CERR | Distrito Federal | 15°56’S/47°55’W | Gama – Lago Paranoá | Riparian | 10 | 3.2 | 1412 | 35 |
| Veloso (2011) PhD-Diss. Univ. Federal de Lavras – Brazil. | CERR | Minas Gerais | 15°40’S/44°38’W | Pandeiros – São Francisco | Riparian | 5 | 0.7 | 463 | 52 |
| Veloso (2011) PhD-Thesis Univ. Federal de Lavras – Brazil. | CERR | Minas Gerais | 15°36’S/44°42’W | Pandeiros – São Francisco | Riparian | 5 | 0.7 | 994 | 59 |
| Veloso (2011) PhD-Thesis Univ. Federal de Lavras – Brazil. | CERR | Minas Gerais | 15°30’S/44°45’W | Pandeiros – São Francisco | Riparian | 5 | 0.7 | 759 | 108 |
| Veloso (2011) PhD-Thesis Univ. Federal de Lavras – Brazil. | CERR | Minas Gerais | 15°07’S/45°12’W | Pandeiros – São Francisco | Riparian | 5 | 0.7 | 446 | 54 |
| Veloso (2011) PhD-Thesis Univ. Federal de Lavras – Brazil. | CERR | Minas Gerais | 15°17’S/45°00’W | Pandeiros – São Francisco | Riparian | 5 | 0.7 | 301 | 41 |
| Veloso (2011) PhD-Thesis Univ. Federal de Lavras – Brazil. | CERR | Minas Gerais | 15°25’S/44°50’W | Pandeiros – São Francisco | Riparian | 5 | 0.7 | 688 | 77 |
| Teixeira & Rodrigues (2006) *Acta Bot Bras* 20: 803-813. | CERR | São Paulo | 20°24’S/47°24’W | Rib. dos Cristais – Rio Grande | Swamp | 4.8 | - | 360 | 53 |
| Cardoso-Leite *et al*. (2004) *Rev Inst Flor São Paulo* 16: 31-41. | CERR | São Paulo | 22°10’S/47°20’W | Rib. Claro - Turvo – Rio Grande | Riparian | 6.4 | - | 163 | 40 |
| Silva-Junior (2005) *Cerne* 11: 147-158. | CERR | Distrito Federal | 22°10’S/47°20’W | Córrego do Pitoco – Lago Paranoá | Riparian | 5 | - | 1000 | 99 |
| Sampaio *et al*. (2000) *Acta Bot Bras* 14: 197-214. | CERR | Distrito Federal | 15°56’S/48°02’W | Riacho Fundo – S. Bartolomeu | Riparian | 5 | 0.78 | 1228 | 126 |
| Sampaio *et al*. (2000) *Acta Bot Bras* 14: 197-214. | CERR | Distrito Federal | 15°52’S/48°00’W | Riacho Fundo – S. Bartolomeu | Riparian | 5 | 1.58 | 1831 | 150 |
| Fontes & Walter (2011) *Rev Bras Bot* 34: 145-158. | CERR | Distrito Federal | 15°54’S/48°00’W | Riacho Fundo – S. Bartolomeu | Swamp | 3 | 0.8 | 2728 | 59 |
| Santiago *et al*. (2005) *Sci For Piracicaba* 67: 64-77. | CERR | Distrito Federal | 15°56’S/47°53’W | Córrego do Pitoco – Lago Paranoá | Riparian | < 5 | 1.0 | 2718 | 118 |
| Vilela *et al*. (1998) CEMIG/UFLA/FAEPE (Ed.), Belo Horizonte – Brazil. | CERR | Minas Gerais | 19°09’S/50°39’W | Parnaíba – São Francisco | Riparian | 5 | 1.12 | 1378 | 60 |
| Vilela *et al*. (1999) *Rev Árvore 23*: 423-433. | CERR | Minas Gerais | 19°22’S/45°19’W | Rio Grande | Riparian | 5 | 0.9 | 1512 | 121 |
| Rodrigues *et al*. (2010) *Hoehnea* 37: 87-105. | CERR | Minas Gerais | 18°47’S/48°08’W | Araguari - Parnaíba | Riparian | 4.8 | 1.1 | 1393 | 89 |
| Nobrega *et al*. (2007) *Sci For Piracicaba* 75: 51-63. | CERR | São Paulo | 21°31’S/47°55’W | Mogi Guaçu – Rio Grande | Riparian | 5 | 0.6 | 702 | 25 |
| Nobrega *et al*. (2007) *Sci For Piracicaba* 75: 51-63. | CERR | São Paulo | 21°31’S/47°55’W | Mogi Guaçu – Rio Grande | Riparian | 5 | 0.6 | 716 | 35 |
| Brito *et al*. (2008) *Acta Amazônica* 38: 379-386. | CERR | Tocantins | 10°48’S/49°37’W | Formoso - Tocantins | Riparian | 4.8 | 1.0 | 665 | 49 |
| Guarino & Walter (2005) *Acta Bot Bras* 19: 431-442. | CERR | Distrito Federal | 15°35’S/48°10’W | Riacho Fundo – S. Bartolomeu | Riparian | 3 | 0.8 | 3030 | 60 |
| Guarino & Walter (2005) *Acta Bot Bras* 19: 431-442. | CERR | Distrito Federal | 15°55’S/48°02’W | Riacho Fundo – S. Bartolomeu | Riparian | 3 | 0.8 | 3058 | 53 |
| Oliveira & Felfili (2005) *Acta Bot Bras* 19: 801-811. | CERR | Distrito Federal | 15°57’S/47°56’W | Gama – Lago Paranoá | Riparian | 1 -5 | 3.2 | 6407 | 104 |
| De Paula *et al.* (1996) *Cerne* 2: 91-105. | CERR | Goiás | 15°48’S/48°21’W | Descoberto | Riparian | 5 | 1.0 | 2361 | 117 |
| Silva Júnior (2004) *Rev Árvore* 28: 419-428. | CERR | Distrito Federal | 15°56’S/47°56’W | Gama – Lago Paranoá | Riparian | 5 | - | 1000 | 110 |
| De Paula *et al*. (1993) *Pesq Agropec Bras* 28: 143-152. | CERR | Distrito Federal | 15°37’S/47°48’W | Riacho Fundo – S. Bartolomeu | Riparian | 5 | 1.0 | 586 | 73 |
| Pinto & Oliveira-Filho (1999) *Rev Bras Bot* 22: 53-67. | CERR | Mato Grosso | 15°20’S/55°50’W | Córrego Coxipozinho – Cuiabá | Riparian | 4.8 | 1.08 | 1363 | 148 |
| Teixeira & Assis (2009) *Acta Bot Bras* 23: 843-853. | CERR | São Paulo | 20°25’S/47°26’W | R. dos Cristais – Rio Grande | Riparian | 3.2 | 0.79 | 2036 | 61 |
| Carvalho *et al.* (2005) *Rev Bras Bot* 28: 329-345. | CERR | Minas Gerais | 18°05’S/45°10’W | São Francisco | Riparian | 5 | 1.08 | 1449 | 117 |
| Miguel (2008) Master-Diss. Univ. do Estado do Mato Grosso, Cáceres – Brazil. | CERR | Mato Grosso | 14°41’S/52°20’W | Paraguai | Riparian | 5 | 0.47 | 503 | 77 |
| Miguel (2008) Master-Diss. Univ. do Estado do Mato Grosso, Cáceres – Brazil. | CERR | Mato Grosso | 14°41’S/52°20’W | Paraguai | Riparian | 5 | 0.47 | 406 | 88 |
| Miguel (2008) Master-Diss. Univ. do Estado do Mato Grosso, Cáceres – Brazil. | CERR | Mato Grosso | 14°41’S/52°20’W | Paraguai | Riparian | 5 | 0.47 | 507 | 71 |
| Güntzel *et al*. (2011) *Acta Bot Bras* 25: 586-592. | CERR | Mato Grosso do Sul | 18°27’S/54°43’W | C. Criminoso – Taquari | Riparian | 6.4 | 0.6 | 327 | 49 |
| Oliveira-Filho (1989) *Acta Bot Bras* 3: 91-112. | CERR | Mato Grosso | 15°21’S/55°49’W | C. da Paciência - Cuiabá | Riparian | 2.9 | 0.2 | 934 | 89 |
| Barbosa *et al*. (2011) *Rev Árvore* 35: 457-471. | CERR | Mato Grosso | 12°19’S/50°44’W | Araguaia | Riparian | 5 | 1.0 | 2177 | 45 |
| Barbosa et al. (2011) Rev Árvore 35: 457-471. | CERR | Mato Grosso | 12°19’S/50°44’W | Araguaia | Riparian | 5 | 1.0 | 1501 | 33 |
| Carvalho *et al*. (1999) *Rev Árvore* 23: 311-320. | CERR | Minas Gerais | 19°09’S/50°39’W | Parnaíba | Riparian | 5 | 1.125 | 1378 | 60 |
| Vilela *et al.* (1999) *Rev Árvore* 23: 423-433. | CERR | Minas Gerais | 19°59’S/47°36’W | Rio Grande | Riparian | 5 | 1.125 | 2188 | 136 |
| Battilani *et al.* (2005) *Acta Bot Bras* 19: 597-608. | AF | Mato Grosso do Sul | 21°24’S/56°22’W | Rio da Prata – Miranda | Riparian | 3.2 | 0.9 | 661 | 66 |
| Donadio *et al*. (2009) *Rev Inst Flor São Paulo* 21: 1-17. | AF | São Paulo | 21°21’S/48°31’W | C. Rico - Tietê | Riparian | 5 | 0.3 | 420 | 54 |
| Marques *et al*. (2003) *Acta Bot Bras* 17: 545-506. | AF | São Paulo | 22°16’S/48°06’W | Jacaré-Pepira - Tietê | Riparian | 5 | 0.36 | 735 | 41 |
| Teixeira *et al*. (2011) *Aquat Bot* 94: 17-23. | AF | São Paulo | 22°15’S/47°40’W | Tietê | Swamp | 3.2 | 0.6 | 5124 | 37 |
| Metzger *et al.* (1997) *Plant Ecol* 133: 135-152. | AF | São Paulo | 22°10’S/48°20’W | Jacaré-Pepira - Tietê | Riparian | 3 | - | 5472 | 222 |
| Pinto *et al*. (2005) *Rev Árvore* 29: 775-793. | AF | Minas Gerais | 21°12’S/45°01’W | R. Santa Cruz – Rio Grande | Riparian | 5 | 2.4 | 4764 | 52 |
| Dias *et al.* (1998) *Rev Bras Bot* 21: 183-195. | AF | Paraná | 24°31’S/50°52’W | Rio Tibagi – Paranapanema | Riparian | 5 | 1.0 | 1594 | 127 |
| Oliveira-Filho *et al*. (1994) *Rev Bras Bot* 17: 67-85. | AF | Minas Gerais | 21°19’S/44°59’W | C. Vilas Boas – Rio Grande | Riparian | 4.9 | 0.48 | 1045 | 119 |
| Campos & De Souza (2002) *Braz Arqu Biol Technol* 45: 137-149. | AF | Paraná | 22°45’S/53°15’W |  | Riparian | 4.8 | 0.89 | 825 | 21 |
| Fagundes *et al.* (2007) *Acta Bot Bras* 21: 65-78. | AF | Minas Gerais | 20°41’S/46°22’W | Rio Grande | Riparian | 5 | 0.48 | 525 | 50 |
| Fagundes *et al*. (2007) *Acta Bot Bras* 21: 65-78. | AF | Minas Gerais | 20°40’S/46°27’W | Rio Grande | Riparian | 5 | 0.52 | 286 | 39 |
| Borghi *et al*. (2004) *Cad Biodivers* 4: 9-18. | AF | Paraná | 22°36’S/52°52’W | Paranapanema | Riparian | 5 | 0.75 | 1146 | 85 |
| Sobrinho *et al*. (2009) *Floresta, Curitiba* 39: 793-805. | AF | Minas Gerais | 20°45’S/42°52’W | S. Bartholomeu - Doce | Riparian | 3 | 0.5 | 375 | 58 |
| Neto *et al.* (2003) *Rev Arvore* 27: 561-574. | AF | Minas Gerais | 20°48’S/42°52’W | Doce | Riparian | 3.2 | 0.175 | 377 | 48 |
| Corino (2006) Master-Diss. Univ. Est. de Maringá – Brazil. | AF | Paraná | 22°57’S/52°02’W | Rio Pirapó – Paranapanema | Riparian | 4.8 | 1.0 | 1375 | 72 |
| Romagnolo & Souza (2000) *Acta Bot Bras* 14: 163-174 | AF | Mato Grosso do Sul | 22°43’S/53°15’W | Paraná | Riparian | 4.8 | 0.95 | 777 | 40 |
| Campos et al. (2000) *Braz. Arch Biol Technol* 43: 185-194. | AF | Mato Grosso do Sul | 22°43’S/53°18’W | Paraná | Riparian | 4.8 | 0.54 | 795 | 47 |
| Ramos *et al*. (2012) *Global Scence Technol, Rio Verde* 5: 133-149. | AF | Minas Gerais | 20°27’S/43°45’W | Rio Garcia - Doce | Riparian | 4.8 | 0.56 | 1258 | 119 |
| Oliveira (2001) Master-Diss. Univ. Federal do Paraná, Curitiba – Brazil. | AF | Paraná | 25°09’S/49°58’W | Tibagi – Paranapanema | Riparian | 3.2 | 0.11 | 1184 | 14 |
| Lima et al. (2012) *Ambiência Guarapuava* 8: 229-244. | AF | Paraná | 25°19’S/51°23’W | Rio das Pedras - Iguaçu | Riparian | 4.8 | 0.28 | 484 | 32 |
| Iurk (2008) Master-Diss. Univ. Federal do Paraná, Curitiba – Brazil. | AF | Paraná | 25°36’S/50°01’W | Iguaçu | Riparian | 4.8 | 2.5 | 1165 | 35 |
| Carvalho (2012) Master-Diss.Instituto Agronômico Campinas – Brazil. | AF | São Paulo | 22°48’S/47°03’W | C. Campinas - Tietê | Riparian | 3 | 0.25 | 971 | 80 |
| Van den Berg & Oliveira-Filho (2000) *Rev Bras Bot* 23: 231-253. | AF | Minas Gerais | 21°21’S/44°36’W | Rio Grande | Riparian | 5 | 0.84 | 2145 | 62 |
| Ivanauskas *et al*. (1997) *Rev Bras Bot* 20: 139-153. | AF | São Paulo | 23°17’S/48°38’W | Paranapanema | Riparian | 4.8 | 1.0 | 1310 | 39 |
| Bertani *et al*. (2001) *Rev Bras Bot* 24: 11-23. | AF | São Paulo | 22°26’S/47°43’W | Passa Cinco - Tietê | Riparian | 5 | 0.785 | 1395 | 105 |
| Souza *et al*. (2003) *Rev Arvore* 27: 185-206. | AF | Minas Gerais | 21°18’S/44°20’W | Capivari – Rio Grande | Riparian | 5 | 1.12 | 1599 | 140 |
| Vilela et al. (2001) *Cerne* 6: 41-54. | AF | Minas Gerais | 21°29’S/44°22’W | Rio Grande | Riparian | 5 | 0.97 | 1535 | 111 |
| Vilela *et al*. (2001) *Cerne* 6: 41-54. | AF | Minas Gerais | 21°29’S/44°22’W | Rio Grande | Riparian | 5 | 0.63 | 203 | 8 |
| Pasidora (2003) Master-Diss. Univ. Federal do Paraná, Curitiba-Brazil. | AF | Paraná | 25°35’S/49°24’W | Iguaçu | Riparian | 3.2 | 0.1 | 3250 | 22 |
| Pasidora (2003) Master-Diss. Univ. Federal do Paraná, Curitiba-Brazil. | AF | Paraná | 25°35’S/49°24’W | Iguaçu | Riparian | 3.2 | 0.1 | 3040 | 23 |
| Carvalho *et al.* (2000) *Acta Bot Bras* 14: 37-55. | AF | Minas Gerais | 19°26’S/43°14’W | Doce | Riparian | 5 | 0.79 | 2430 | 216 |
| Barddal *et al.* (2004) *Ciência Florestal Santa Maria* 14: 37-50. | AF | Paraná | 25°35’S/49°20’W | Barigüi - Iguaçu | Riparian | 4.8 | 0.2 | 507 | 29 |
| Bianchini *et al*. (2003) *Acta Bot Bras* 17: 405-419. | AF | Paraná | 23°27’S/51°15’W | R. dos Apertados - Paranapanema | Riparian | 4.8 | 0.5 | 912 | 64 |
| Botrel *et al*. (2002) *Rev Bras Bot* 25: 195-213. | AF | Minas Gerais | 21°24’S/44°55’W | Rio Grande | Riparian | 5 | 1.0 | 2683 | 135 |
| Costa Filho *et al.* (2006) *Braz Arch Biol Technol* 49: 785-798. | AF | Paraná | 22°41’S/52°55’W | Paranapanema | Riparian | 4.8 | 1.2 | 1487 | 63 |
| Costa Filho *et al.* (2006) *Braz Arch Biol Technol.* 49: 785-798. | AF | Paraná | 22°41’S/52°55’W | Paranapanema | Riparian | 4.8 | 1.05 | 1146 | 85 |
| Prata *et al*. (2011) *Rev Bras Bot* 34: 159-168. | AF | São Paulo | 22°23’S/47°32’W | Ribeirão Claro - Piracicaba | Riparian | 3.2 | 0.44 | 1697 | 22 |
| Toniato *et al.* (1998) *Rev Bras Bot* 21: 197-210. | AF | São Paulo | 22°49’S/47°06’W | Piracicaba | Riparian | 3.2 | 0.2 | 955 | 55 |
| Silva *et al*. (1995) *Acta Bot Bras* 9: 289-302. | AF | Paraná | 24°01’S/50°41’W | Tibagi - Paranapanema | Riparian | 5 | 1.0 | 1560 | 125 |
| Giehl (2007) Master-Diss. Univ. Federal do Rio Grande do Sul, Porto Alegre – Brazil. | AF | Rio Grande do Sul | 27°09’S/53°53’W | Uruguai | Riparian | 4.8 | 1.0 | 1615 | 83 |
| Camargos *et al.* (2008) *Acta Bot Bras* 22: 75-82. | AF | Minas Gerais | 19°48’S/42°38’W | Doce | Riparian | 4.8 | 1.0 | 1187 | 35 |
| Carvalho *et al*. (2006) *Rodriguesia* 57: 503-518. | AF | Rio de Janeiro | 22°33’S/42°15’W | São João | Riparian | 10 | 0.36 | 361 | 21 |
| Carvalho *et al.* (2006) *Rodriguesia* 57: 503-518. | AF | Rio de Janeiro | 22°33’S/42°15’W | São João | Riparian | 10 | 0.36 | 267 | 20 |
| Rocha *et al.* (2005) *Rev Bras Bot* 28: 203-218. | AF | Minas Gerais | 21°09’S/45°28’W | Maribondo – Rio Grande | Riparian | 5 | 0.32 | 581 | 100 |
| Sztutman & Rodrigues (2002) *Rev Bras Bot* 25: 161-176. | AF | São Paulo | 24°40’S/47°48’W | Ribeira do Iguape | Swamp | 4.8 | 0.3 | 520 | 46 |
| Teixeira & Assis (2005) *Rev Bras Bot* 28: 467-476. | AF | São Paulo | 22°21’S/47°28’W | Piracicaba | Swamp | 4.8 | 0.45 | 1651 | 49 |
| Loures *et al.* (2007) *Acta Bot Bras* 21: 885-896. | AF | Minas Gerais | 22°05’S/46°21’W | Rio Pardo – Rio Grande | Riparian | 5 | 1.0 | 2982 | 110 |
| Nascimento *et al.* (2011) *Ciência Florestal Santa Maria* 21: 209-218. | AF | Santa Catarina | 27°48’S/50°06’W | Lambedor - Canoas | Riparian | 5 | 0.5 | 1047 | 67 |
| Pedreira & Souza (2011) *Ciência Florestal Santa Maria* 21: 663-675. | AF | Minas Gerais | 20°25’S/43°30’W | C. do Manso | Swamp | 4.8 | 0.42 | 1136 | 53 |
| Silva et al. (2009) *Rev Bras Bot* 32: 283-497. | AF | Minas Gerais | 22°05’S/45°48’W | Sapucaí – Rio Grande | Riparian | 5 | 1.08 | 1911 | 82 |
| Assumpção & Nascimento (2000) *Acta Bot Bras* 14: 301-315. | AF | Rio de Janeiro | 21°44’S/41°02’W | - | Restinga | 2.5 | 0.09 | 380 | 61 |
| Guedes *et al.* (2006) *Acta Bot Bras* 20: 299-311. | AF | São Paulo | 23°51’S/46°08’W | - | Restinga | 3.2 | 0.48 | 476 | 65 |
| Lima *et al*. (2011) *Acta Bot Bras* 25: 633-645. | AF | São Paulo | 25°04’S/47°55’W | - | Restinga | 4.8 | 10.24 | 15014 | 116 |
| Menezes *et al*. (2010) *Acta Bot Bras* 24: 825-839. | AF | Rio de Janeiro | 23°02’S/43°35’W | - | Restinga | 2.5 | 0.5 | 1223 | 57 |
| Oliveira *et al*. (2009) *Rev Bras Ciências Agrárias* 4: 167-172. | AF | Pernambuco | 07°28’S/35°09’W | Capibaribe Mirim - Goiana | Riparian | 4.8 | 0.26 | 282 | 32 |
| Meira-Neto *et al*. (1998) *Rev Arvore* 22: 179-184. | AF | Minas Gerais | 20°30’S/43°01’W | Rio Piranga - Doce | Riparian | 4.8 | 0.12 | 200 | 55 |
| Santos Júnior (2011) Diss. Univ. do Extremo Sul Catarinense, Criciúma – Brazil. | AF | Rio Grande do Sul | 29°37’S/50°04’W | - | Restinga | 5 | 0.5 | 1027 | 38 |
| Veiga *et al*. (2003) *Acta Scientiarum Agronomy Maringá* 25: 519-525. | AF | Paraná | 23°10’S/52°00’W | Pirapó - Paranapanema | Riparian | 5 | 0.75 | 922 | 48 |
| Nascimento *et al*. (2003) *Rev Bras Bot* 26: 271-287. | CAAT | Pernambuco | 09°02’S/40°14’W | São Francisco | Riparian | 3 | 1.4 | 2234 | 48 |
| Farias & Castro (2004) *Acta Bot Bras* 178: 949-963. | CAAT | Piauí | 04°52’S/42°03’W | Parnaíba | Riparian | 3 | - | 402 | 46 |
| Farias & Castro (2004) *Acta Bot Bras* 178: 949-963. | CAAT | Piauí | 04°52’S/42°04’W | Parnaíba | Riparian | 3 | - | 398 | 44 |
| Matos & Felfili (2010) *Acta Bot Bras* 24: 483-496. | CAAT | Piauí | 04°06’S/41°42’W | Parnaíba | Riparian | 5 | 0.56 | - | 75 |
| Andrade *et al.* (2006) *Rev Bras Ciências Agrárias* 1: 31-40. | CAAT | Paraíba | 06°58’S/35°42’W | - | Swamp | 5 | 1.68 | 2659 | 67 |
| Trovão *et al*. (2010) *Rev Caatinga* 23: 78-86. | CAAT | Paraíba | 07°31’S/35°59’W | R. Bodocongó – Rio Paraíba | Riparian | 3 | 0.24 | 357 | 17 |
| Ferraz *et al*. (2006) *Acta Bot Bras* 20: 125-134. | CAAT | Pernambuco | 08°35’S/38°34’W | Riacho do Navio – São Francisco | Riparian | 10 | 0.96 | - | 24 |
| Aranha *et al.* (2010) *Rev Inst Flor* 22: 1-14. | CAAT | Bahia | 09°09’S/40°22’W | São Francisco | Riparian | 3 | 0.54 | 853 | 32 |
| Queiroz Matos (2009) Master-Diss. Universidade de Brasília, Brasília-Brazil. | CAAT | Piauí | 04°02’S/41°40’W | Piracuruca - Parnaíba | Riparian | 5 | 0.56 | 642 | 75 |
| Santos-Filho (2009) PhD-Diss. Universidade Federal Rural de Pernambuco, Recife-Brazil. | CAAT | Piauí | 02°53’S/41°45’W | Parnaíba | Restinga | 3 | - | 200 | 12 |
| Santos-Filho (2009) PhD-Diss. Universidade Federal Rural de Pernambuco, Recife-Brazil. | CAAT | Piauí | 02°53’S/41°45’W | Parnaíba | Restinga | 3 | - | 200 | 18 |
| Santos-Filho (2009) PhD-Diss. Universidade Federal Rural de Pernambuco, Recife-Brazil. | CAAT | Piauí | 02°53’S/41°45’W | Parnaíba | Restinga | 3 | - | 200 | 23 |
| Waechter & Jarenkow (1997) *Biotemas* 11: 45-69. | PM | Rio Grande do Sul | 32°40’S/52°30’W | - | Restinga / Swamp | 10 | - | 120 | 12 |
| Scherer *et al.* (2005) *Acta Bot Bras* 19: 717-726. | PM | Rio Grande do Sul | 30°22’S/51°00’W | - | Restinga / Swamp | 5 | 1.02 | 1023 | 31 |
| Soares & Ferrer (2009) *Biotemas* 22: 47-55. | PM | Rio Grande do Sul | 31°35’S/53°02’W | Piratini | Riparian | 5 | 0.5 | 702 | 40 |
| Saraiva (2011) *Biotemas* 24: 49-58. | PM | Rio Grande do Sul | 31°26’S/53°46’W | Jaguarão | Riparian | 5 | 0.25 | 725 | 27 |
| Ceconi (2010) PhD.-Diss. Universidade Federal de Santa Maria – Brazil. | PM | Rio Grande do Sul | 29°45’S/53°43’W | Vacacaí-mirim Jacuí | Riparian | Height > 30 cm | 0.36 | 2483 | 64 |
| Avila et al. (2011) *Ciencia Florestal Santa Maria* 21: 251-260. | PM | Rio Grande do Sul | 28°17’S/53°52’W | Ijuí - Uruguai | Riparian | 4.8 | 0.32 | 463 | 38 |
| Marchi & Jarenkow. (2008) Iheringia, *Sér Bot Porto Alegre* 63: 241-248. | PM | Rio Grande do Sul | 31°01’S/51°56’W | Camaquã | Riparian | 5 | 1.0 | 2179 | 21 |
| Budke *et al*. (2007*) Plant Ecol* 189: 187-200. | PM | Rio Grande do Sul | 30°01’S/52°47’W | Botucaraí - Jacuí | Riparian | 4.8 | 1.0 | 1527 | 30 |
| Budke *et al*. (2008) Flora 203: 162-174. | PM | Rio Grande do Sul | 30°01’S/52°47’W | Botucaraí - Jacuí | Riparian | 4.8 | 1.0 | 1904 | 48 |
| Leão (2009) Diss. Pontifícia Univ. Católica do Rio Grande do Sul, Uruguaiana – Brazil. | PM | Rio Grande do Sul | 29°46’S/56°57’W | Arroio Imbaá - Uruguai | Riparian | 4.8 | 0.45 | 821 | 39 |
| Budke *et al*. (2004) Acta Bot Bras 18: 581-589. | PM | Rio Grande do Sul | 29°45’S/53°45’W | Passo das Tropas - Jacuí | Riparian | 4.8 | 1.0 | 2195 | 57 |
| Dorneles & Waechter (2004) *Acta Bot Bras* 18: 815-824. | PM | Rio Grande do Sul | 31°29’S/50°46’W | Passo das Tropas - Jacuí | Restinga / Swamp | 5 | 1.0 | 3479 | 21 |
| Lindenmaier & Budke (2006) *Pesquisas Botânica* 57: 193-216. | PM | Rio Grande do Sul | 30°00’S/52°48’W | Rio Jacuí | Riparian | 4.8 | 1.0 | 1097 | 49 |
