# Supplementary figures and images for: The Brazilian freshwater wetscape: Changes in tree community diversity and composition on climatic and geographic gradients
Source: PLoS One. 2017 Apr 10;12(4):e0175003. doi: 10.1371/journal.pone.0175003 (PMC5386251; doi:10.1371/journal.pone.0175003)

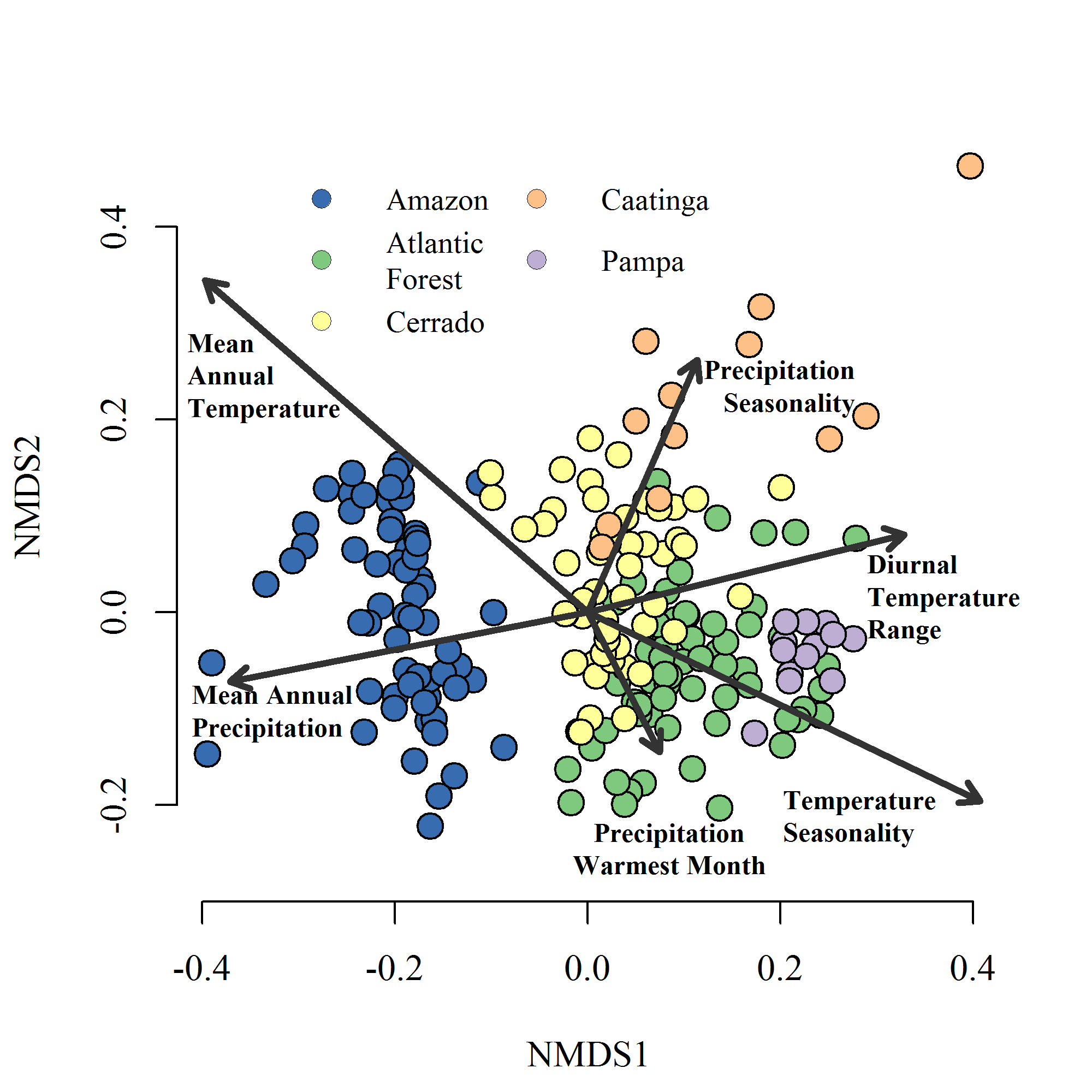

Supplement: S1 Fig — The solution was optimized for two dimensions and rotated to principal components (see Fig 2). The main distinction with the principal components configuration is the relative position of Caatinga sites, which are more outlying in the NMDS. Fitted vectors are of WorldClim climate data [41]. (TIFF) [file pone.0175003.s004.tiff]

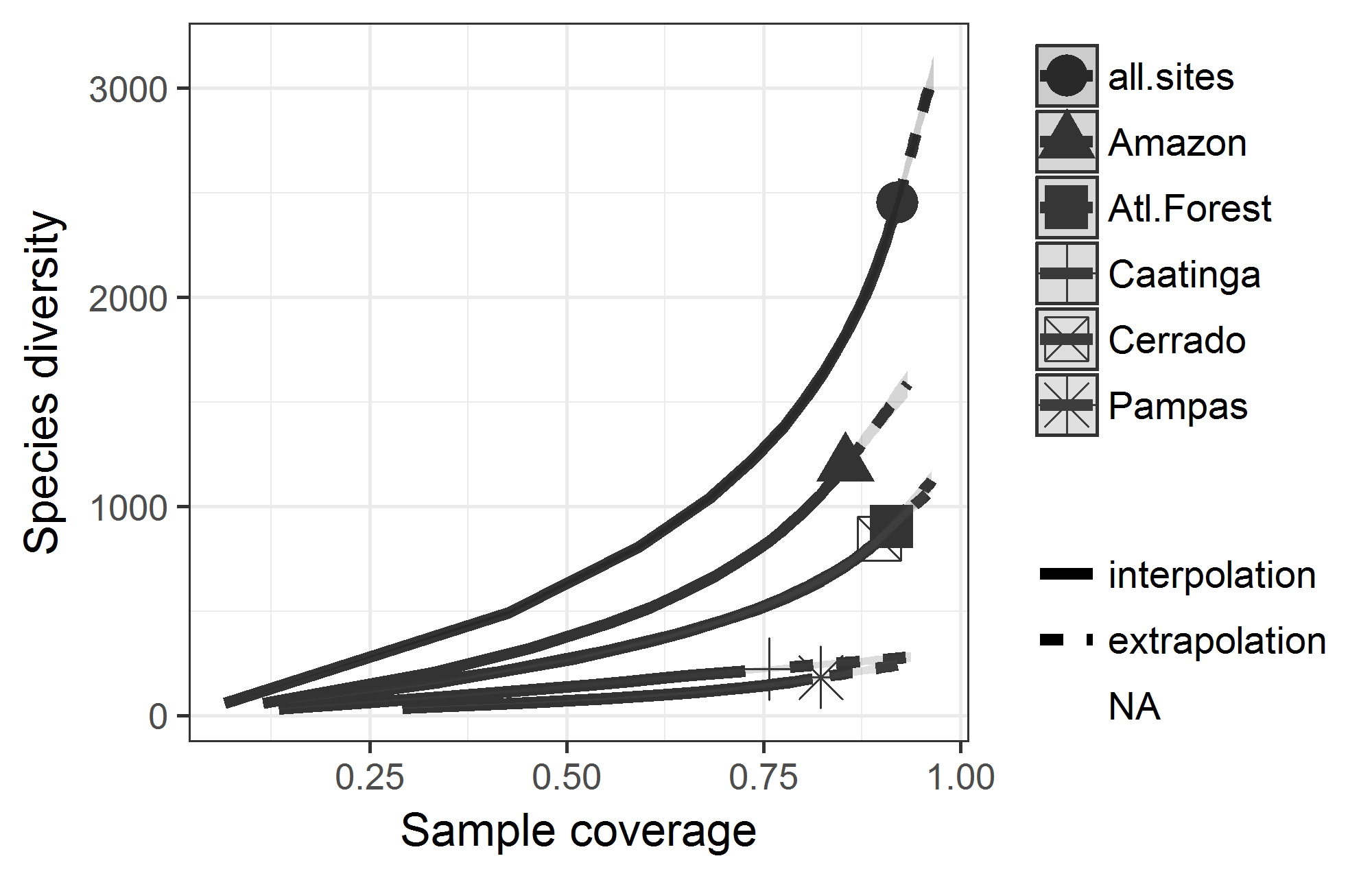

Supplement: S2 Fig — We used the function iNEXT from the ‘iNEXT’ package [56] with the settings iNEXT(x, q = 0, datatype = “incidence_freq”, conf = 0.95). (TIFF) [file pone.0175003.s005.tiff]

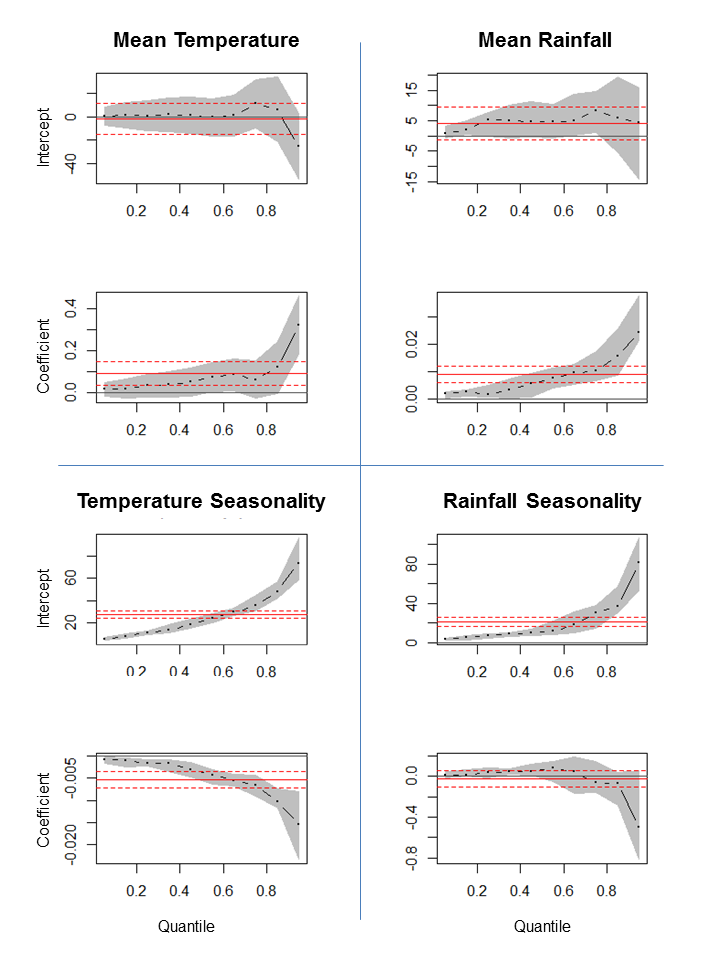

Supplement: S3 Fig — Generally, coefficients are nearly zero for the lower to mid quantiles of the distribution, but rapidly increase at the highest quantiles. The pattern indicates strong climate association only at maximal wetland tree diversity, driven by relatively few sites. (TIF) [file pone.0175003.s006.tif]

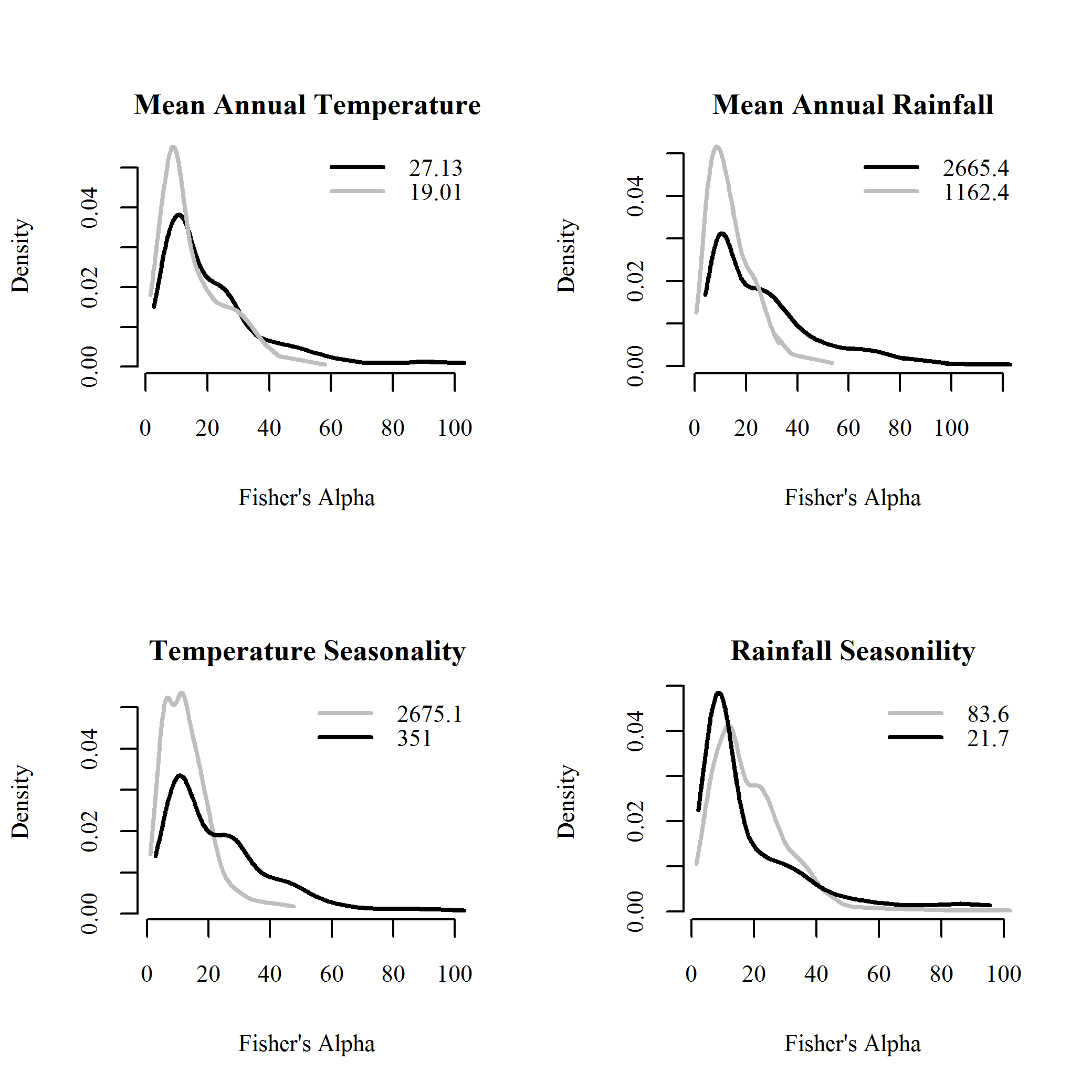

Supplement: S4 Fig — Estimated density functions are based on the corresponding quantile regression solutions (see Fig 4) for all values of tau in 0 to 1. Estimates are presented for the 10th and 90th quantiles of each climate variable, as specified in the legend insets. Mean annual temperature is multiplied by 10, as in the original WorldClim database [41]. (TIFF) [file pone.0175003.s007.tiff]

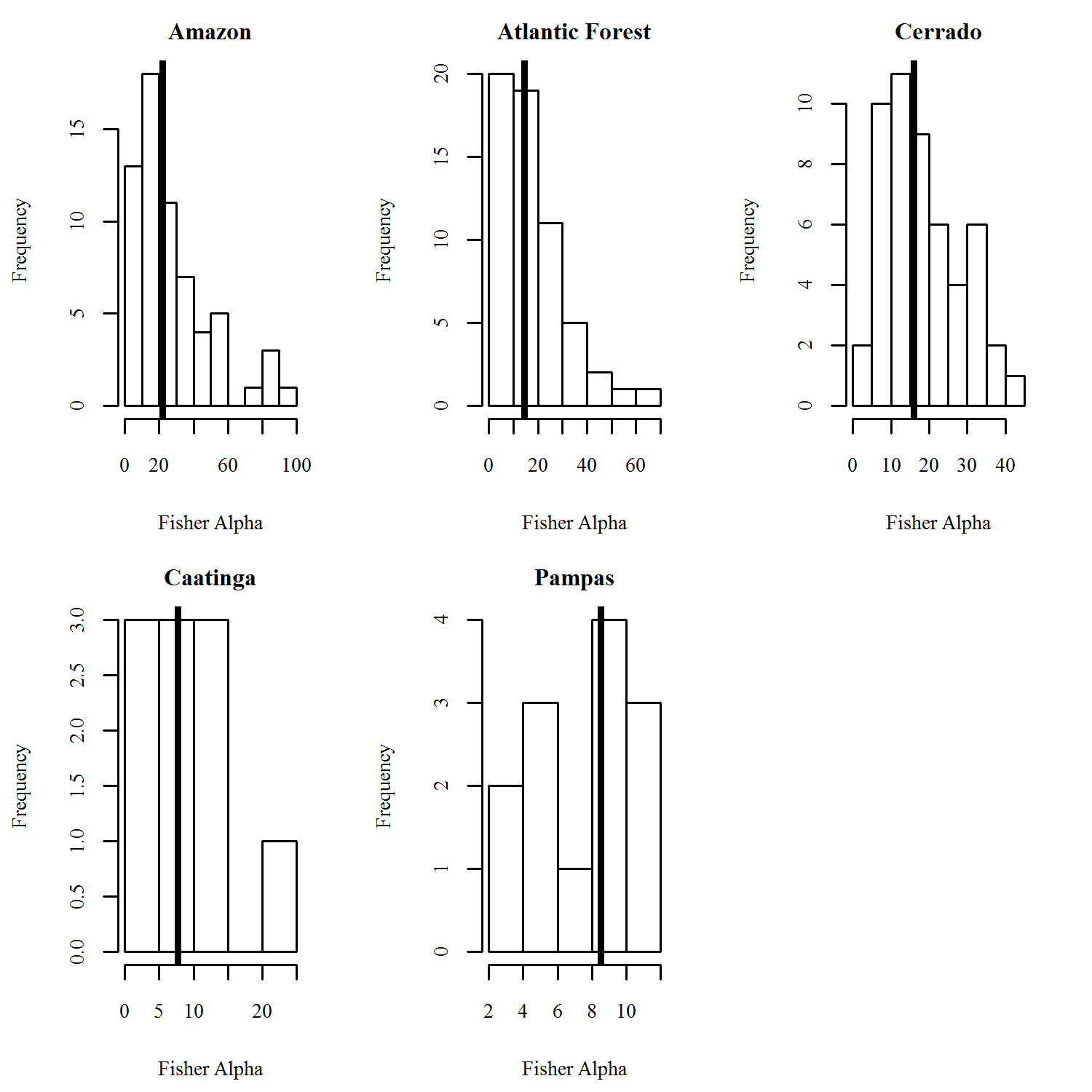

Supplement: S5 Fig — Median values are indicated with a thick vertical line. Note that median values are relatively similar for the three best-sampled biomes (Amazon, Atlantic Forest, and Cerrado). (TIFF) [file pone.0175003.s008.tiff]
